# Role of Mitophagy in Cardiovascular Disease

**DOI:** 10.14336/AD.2019.0518

**Published:** 2020-03-09

**Authors:** Yibo Yang, Tianyi Li, Zhibo Li, Ning Liu, Youyou Yan, Bin Liu

**Affiliations:** Department of Cardiology, The Second Hospital of Jilin University, Changchun 130041, China

**Keywords:** cardiomyocyte, cardiovascular disease, mitochondria, mitophagy

## Abstract

Cardiovascular disease is the leading cause of mortality worldwide, and mitochondrial dysfunction is the primary contributor to these disorders. Recent studies have elaborated on selective autophagy-mitophagy, which eliminates damaged and dysfunctional mitochondria, stabilizes mitochondrial structure and function, and maintains cell survival and growth. Numerous recent studies have reported that mitophagy plays an important role in the pathogenesis of various cardiovascular diseases. This review summarizes the mechanisms underlying mitophagy and advancements in studies on the role of mitophagy in cardiovascular disease.

## 1. Introduction

Owing to the epidemiological shift in the 20^th^ century, infectious disease-related death and disability have decreased; however, they have increased rapidly owing to noninfectious diseases including cardiovascular disease (CVD), which have some of the highest mortality and morbidity rates. After the 1930s, mortalities due to CVD exceeded those due to tumors, tuberculosis, and pneumonia. CVD became a leading cause of mortality in developed countries [1]. CVD includes heart and vascular diseases and is a major public health concern and cause of mortality [2]. The prevalence of CVD has increased notwithstanding improvements in treatment and management methods. Age-standardized mortality of CVD has decreased by 14.5% over the past two decades [3]. Nonetheless, with an increase in the incidence of cardiovascular disease and changes in the disease spectrum, the incidence of CVD and CVD-associated mortality are expected to increase worldwide in future decades. Therefore, prevention and treatment of cardiovascular disease is extremely urgent [4]. In CVD, the final and the most prominent phenomenon is cell death caused by harmful stimuli and aging. Mitochondria provide energy for cellular metabolism and are involved in cell death. They participate in cell proliferation, apoptosis, signal transduction, and calcium homeostasis. Hence, the mitochondrial steady state is essential for cellular function [5-7].

Mitochondria are the most abundant organelles in cardiomyocytes and are very important for the maintenance of normal cell function. Mitophagy is selective autophagy wherein cells eliminate damaged or dysfunctional mitochondria, which is essential for the regulation of mitochondrial homeostasis [8-10]. Mitophagy was first proposed by Lemasters et al. in 2005. Since then, numerous diseases have been associated with mitophagy [11]. Recent studies revealed that mitophagy contributes to the pathophysiology of CVD. Mitophagy regulates cardiovascular activity through several pathways [12-22]. The present review discusses mitophagy and its underlying mechanisms, methods to assess mitophagy and its involvements in CVD.

**Figure 1.**
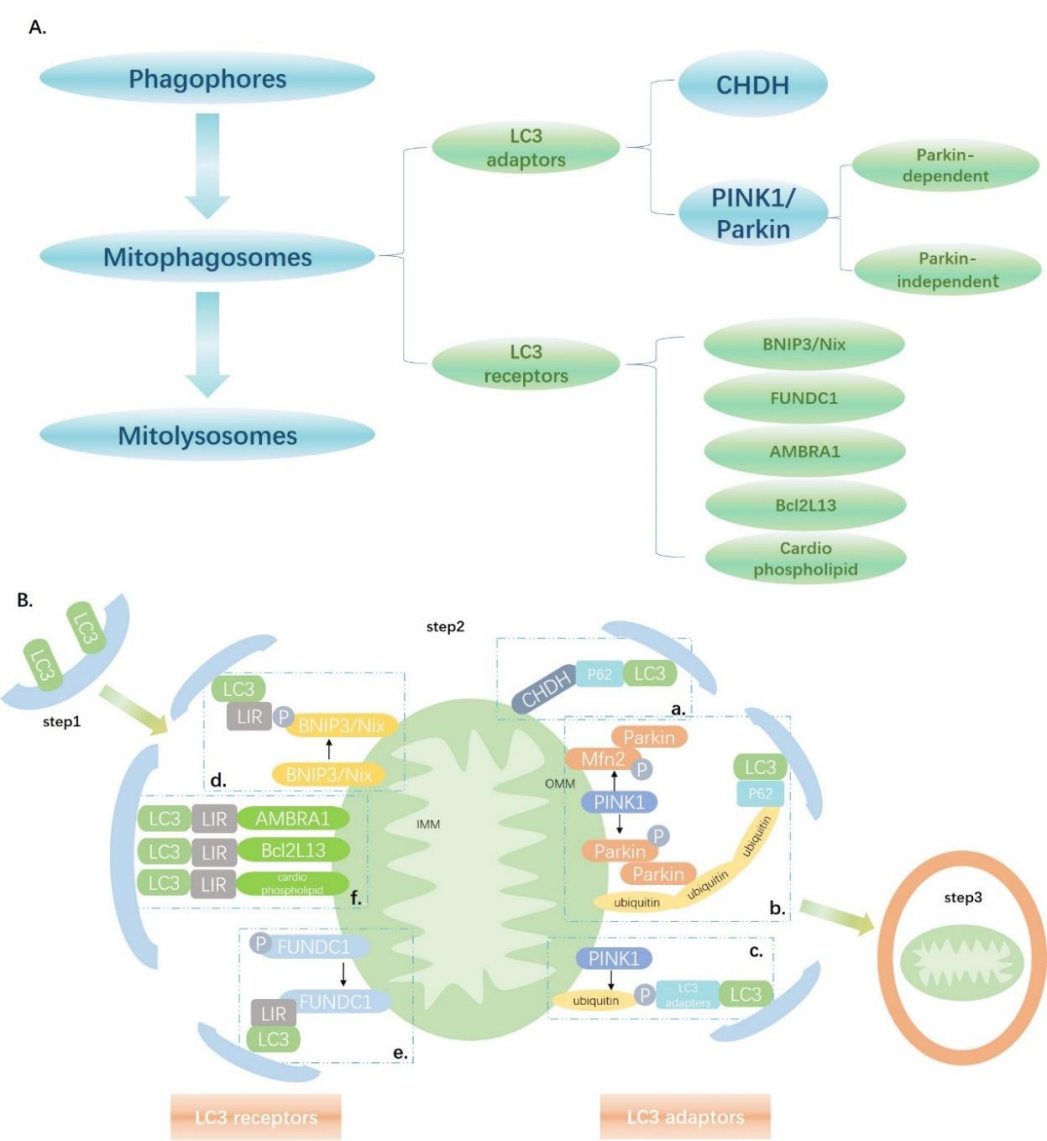
The mechanisms underlying mitophagy. (A) An overview of the mechanisms underlying mitophagy. (B) Step1: Phagophores are formed by the isolated membrane and LC3. Step2: Thereafter, through LC3 adaptors and LC3 receptors, damaged mitochondria can be recognized and form mitophagosomes. The detailed mechanism can be divided into six stages. a. CHDH accumulates on the outer mitochondrial membrane (OMM) and interacts with p62 and binds with LC3. b. PINK1 accumulates on the OMM, phosphorylates Parkin and Mfn2, thus recruiting Parkin to the OMM, and Parkin helps generate ubiquitin chains on the OMM, which can recognize p62 and bind with LC3. c. PINK1 phosphorylates ubiquitin on the OMM and LC3 adapters can bind with it. d. LC3 directly recognizes BNIP3 or Nix through LIR, and phosphorylation of LIR in BNIP3 promotes the interaction between BNIP3 and LC3. e. Dephosphorylation of FUNDC1 restores its ability to interact with LC3 through LIR. f. AMBRA1, Bcl2L13, and cardiophospholipids directly recognize LC3 through LIR. Step3: Mitophagosomes and lysosomes fuse into mitolysosomes.

## 2. Mitophagy

Mitochondria regulate calcium steady state, signal transmission between organelles, and oxidative phosphorylation for ATP biosynthesis. Mitochondrial DNA (mtDNA) is present in the matrix or membrane and does not combine with histones. Therefore, mtDNAs are relatively vulnerable to reactive oxygen species (ROS) and influenced by mutations. Mitochondria are more susceptible to oxidative damage than other organelles [23-25]. Dysfunctional and damaged mitochondria generate excess ROS, which damage proteins, membrane lipids, nucleic acids, adjacent mitochondria and release proapoptotic proteins into the cytosol, eventually causing cell death [25-28]. Hence, prompt and appropriate elimination of damaged or aging mitochondria is vital for normal cell growth. This process is executed by mitophagy [11].

Autophagy involves the degradation of the protoplasm by lysosomes, resulting in macrophage-, microphage-, and chaperone-mediated autophagy. Selective autophagy occurs in response to nutrient deficiency, whereas nonspecific autophagy is caused by cell aging or a response to damaged cell components. Mitophagy is a selective autophagy pathway involving the elimination of abnormal mitochondria and maintains mitochondrial homeostasis [16, 29-32].

### 2.1 Mitophagy and related factors

Mitophagy is divided into three steps: formation of phagocytic vesicles, recognition of damaged mitochondrial components and compartmentation into mitochondrial phagocytic vesicles, and fusion of mitochondrial phagocytic vesicles and lysosomes into mitolysosomes [16, 33, 34](Fig. 1). The mitophagy pathway was first identified in yeast in 2004. Mitochondrial outer membrane protein autophagy-related gene 32 (Atg32) recruits core autophagy molecules to the mitochondria for selective clearance [35-37]; however, no homologs have been reported in the human genome thus far. The subsequent section summarizes the mechanisms underlying mitophagy in accordance with the three aforementioned stages.

### Step 1. Formation of phagocytic vesicles (Phagophores)

Phagocytic vesicles are isolated membranous structures in the endoplasmic reticulum (ER) of mammalian cells. The formation of phagocytic vesicles is regulated by an evolutionarily conserved protein (Atg) system [35, 37-39]. Atg6 recruits other Atg proteins to the isolation membrane, thus warranting the Atg5-Atg12 and LC3-phosphatidylethanolamine conjugation systems. Covalent conjugation between Atg12 and Atg5 resembles ubiquitination, wherein Atg12 is initially activated by Atg7, and then transferred to Atg10. Atg12 is then covalently conjugated with Atg5 at a lysine residue and Atg10 is released. Finally, the Atg12-Atg5 complex interacts non-covalently with Atg16, which then initiates the elongation of the membrane by recruiting LC3-phosphatidylethanolamine [39]. LC3 is a surface molecule on phagocytic vesicles. Phagocytic vesicles are formed by combining LC3-I with phosphatidylethanolamine to produce lipid LC3-II [33, 40, 41].

### Step 2. Recognition of damaged mitochondrial components and compartmentation into mitochondrial phagocytic vesicles (Mitophagosomes)

Mitophagy is a type of selective organellar autophagy in mammalian cells. Identification of phagocytic vesicles and their combination with damaged mitochondria have received increasing attention in studies on mitophagy [33, 42]. Damaged mitochondria are recognized by LC3 adapters or LC3 receptors. First, we address the recognition of impaired mitochondria by LC3 adapters.

#### PINK1 and Parkin

In mammalian cells, PINK1/Parkin-mediated mitophagy is the most extensively characterized mitophagic phenomenon thus far [43]. PINK1 is a serine/threonine kinase with an N-terminal mitochondrial targeting signal [44]. In intact mitochondria, PINK1 is recruited to the mitochondria by translational enzymes on the outer mitochondrial membrane (OMM) and the endometrial complex, anchored within the mitochondrial endometrium, and continuously degraded by protease and peptidase. During mitochondrial depolarization, however, translocation of PINK1 across to the inner mitochondrial membrane (IMM) is inhibited. PINK1 is not degraded but aggregates at the outer membrane [16, 42].

PINK1 utilizes its own ubiquitin-like domain to function like ubiquitin. Activated PINK1 phosphorylates Parkin (a cytoplasmic E3 ubiquitin ligase) at T175, thus activating Parkin, which in turn aggregates in the OMM. However, activated PINK1 also phosphorylates Mfn2, which can then serve as a Parkin receptor in cardiomyocyte mitochondria. This process also induces Parkin aggregation on the OMM [43, 45-47]. Phospho-parkin helps generate ubiquitin chains on the OMM proteins through polyubiquitination, thus amplifying the signal, and these ubiquitin chains serve as LC3 adapter recognition sites, which bind to LC3 and initiate mitophagy [48, 49].

PINK1 may also be recruited in a Parkin-independent manner. PINK1 phosphorylates the mitochondrial ubiquitin on OMM at S65. LC3 adapters such as optineurin and NDP52 recognize the damaged mitochondria and ubiquitin phosphorylated at S65 through the ubiquitin-binding region [48].

#### Choline dehydrogenase (CHDH)

CHDH oxidizes choline to betaine aldehyde in the mitochondria. In normal mitochondria, CHDH is unanchored at both the OMM and IMM. In depolarized mitochondria, CHDH accumulates on the OMM and its FAD/NAD-binding domain 1 interacts with the Phox and Bem1 domains in cytoplasmic p62/SQSTM1, thus recruiting p62/SQSTM1 at damaged mitochondria and binding with LC3 [42, 50].

In summary, the impaired mitochondrial recognition process mediated by the LC3 adapter is divided into the ubiquitin-associated type (PINK1/Parkin) and the ubiquitin-independent type (CHDH). Five LC3 adapters have been identified including OPTN, p62/SQSTM1, NBR1, NDP52, and TAX1BP, which mediate the recognition and phagocytosis of damaged mitochondria by joining LC3-coated phagocytic vesicles to label damaged mitochondria [33, 42]. Further, we discuss the LC3 reporter-mediated recognition of impaired mitochondria.

#### Nix and BNIP3

Nix and BNIP3 are OMM proteins that interact with LC3 or its homologs through the LC3-interacting region (LIR) to form mitophagy receptors. BNIP3 and Nix are hypoxia-induced tail-anchored proteins that bind to the OMM via the carboxyl transmembrane domain. The N-terminal extends into the cytoplasm and is linked to LC3-related molecules[51-53]. BNIP3-dependent mitophagy requires drp1 to induce mitochondrial division. Inhibition of this process or drp1 knockout blocks hypoxia-induced mitophagy. Nix protein levels increase after erythrocyte maturation. Nix knockout inhibits the loss of the mitochondrial membrane potential and the isolation of mitochondrial autophagosomes. Nix-dependent mitophagy is induced by the loss of membrane potential caused by carbonyl cyanide m-chlorophenylhydrazone treatment. Therefore, Nix-mediated loss of membrane proteins is important in mitophagy [54].

#### FUNDC1

FUNDC1 is an OMM protein, which integrates with LC3 under hypoxia via its LIR structure. During starvation, mitophagy does not depend on FUNDC1; therefore, it specifically induces hypoxia [55]. FUNDC1 is expressed under normoxic conditions; however, FUNDC1 and LC3 interact under hypoxic conditions upon dephosphorylation at Y18 [56]. Furthermore, FUNDC1 is a mitochondrial receptor for unc-51-like autophagy activating kinase 1 (ULK1), which in turn is translocated to depolarized mitochondria and FUNDC1 phosphorylated at S17 and promotes mitophagy [56]. Phosphoglycerate mutase family member 5 phosphatase is located at the mitochondria and dephosphorylates FUNDC1 at S13 under hypoxic conditions or upon treatment with carbonyl cyanide p-trifluoromethoxy phenylhydrazone. The latter promotes the interaction of the enzyme with LC3 and induces mitophagy [57].

LC3 receptors BNIP3/Nix and FUNDC1 have been extensively characterized in cardiovascular studies. BCL2-like13 (Bcl2L13), autophagy/Beclin 1 regulator 1 (AMBRA1), and cardio phospholipids serve as LC3 receptors during mitophagy [16, 42, 58]. They localize at the OMM thus eliminating the need for mitophagic receptors to mark the damaged mitochondria. Rather, they directly interact with LC3 via the LIR and identify and engulf the damaged mitochondria [42, 48, 50].

### Step 3. Mitochondrial phagocytic vesicles and lysosomes fuse into mitolysosomes

Phagocytic vesicles fuse with damaged mitochondria to form mitolysosomes. Thereafter, various hydrolytic enzymes in the lysosome digest the damaged mitochondrial components, thereby executing the final step of mitophagy. Recent studies on mitophagy primarily focused on the formation, recognition, and interaction of phagocytic vesicles with damaged mitochondria. The detailed mechanism underlying the binding of phagocytic vesicles with lysosomes shall not be described in this review.

### 2.2 Research methods

Discussed herein are several widely used techniques to assess mitophagy.

#### Transmission electron microscopy (TEM)

The most prominent phenomenon in autophagy is the formation of a double-layered membrane around the cytosol and/or organelles. This structure fuses with the lysosome, its contents are released, and a single-layer membrane is formed. Therefore, early and late autophagy are easily distinguished by observing double- and single-membrane structures. Consistent with cell autophagy, early mitophagy potentially results in packaging of the double-membrane structure, which can be readily identified on the basis of its unique structure-mitochondrial crest. During late autophagy, mitochondria are transported to the lysosomes and can be identified from their density or residual structure. TEM is currently the best method to detect mitophagy. It is the "gold standard" method to directly detect autophagy [59, 60]. However, TEM has several limitations. Electron microscopy requires extensive sample preparation. Cell damage potentially resulting from human error interferes with observations. Moreover, mitochondrial structures are difficult to discern during later stages of autophagy. Therefore, this method has low sensitivity.

#### Western blotting

A commonly used method to detect autophagy proteins is western blotting. Autophagy can be detected on the basis of the ratio of the LC3B II/I type marker protein and the extent of P62 degradation [61]. Mitophagy is accompanied by the degradation of several mitochondrial proteins; hence, it may also be evaluated by measuring their levels. Mitochondrial degradation occurs through the autophagy pathway, and cytosolic LC3 protein is converted from type I to type II via conjugation with phosphatidylethanolamine. Therefore, LC3 detection is also a potential method of detecting autophagy. The use of autophagy inhibitors including Bafilomycin A1 (Baf A1) and hydroxychloroquine (HCQ) is also a prominent method of detecting autophagy [41, 62]. Recent studies have reported that the OMM, IMM, and matrix proteins are degraded via mitophagy, whereas certain proteins regulating mitochondrial division and migration are degraded by proteasomes or ATP-dependent protease pathways. TIM23, TOM20, Hsp60, and electron transport chain (ETC)-related proteins are commonly used as mitophagy markers [63]. P62 can be recruited individually, since it is highly ubiquitinated after mitochondrial damage. P62 is involved in numerous signal transduction pathways and its function is complex. Therefore, the role of P62 in mitophagy detection is controversial [64, 65].

#### Fluorescence labeling

GFP-LC3 is often used as an autophagosome marker. Following plasmid transfection, autophagosome formation may be assessed using a fluorescence microscope. Similarly, plasmids containing fluorescent markers or specific antibodies against certain mitochondrial proteins including TOM20 and cytochrome C can be used to label mitochondria. Several commercialized mitochondrial dyes such as MitoTracker and TMRE (Tetramethylrhodamine ethyl ester) are also potentially applicable. In general, mitochondria and autophagosomes/lysosomes can be labeled separately and the colocalization of mitochondria, autophagosomes, and lysosomes may be detected via confocal laser microscopy to detect mitophagy. LC3 fused with fluorescent proteins, such as GFP and RFP, helps observe mitochondrial incorporation into autophagosomes, accompanied with the extension of the isolation membrane through time-lapse microscopic imaging of live cells. GFP fluorescence is attenuated under acidic conditions; hence, GFP-LC3 does not label autolysosomes. However, RFP is resistant to the acidic environment of lysosomes. By harnessing the distinct properties of GFP and RFP, GFP-RFP-tandem tagging LC3 is a potentially useful tool to quantify mitophagy and helps distinguish autophagosomes and autolysosomes. However, this evaluation may overlook the previously reported mitophagy mediated via an LC3-independent mechanism [66-68]. Recently, a mitochondrial probe MitoTimer was developed, which is a red fluorescent protein Ds Red "mutant" and encodes a redox sensitive mitochondrial targeted protein that switches from green to red fluorescence upon oxidation [69]. Therefore, MitoTimer is an ROS-dependent fluorophore. Furthermore, changes in the transition from green to red fluorescence are affected by temperature, oxygen, and light exposure; hence, this assay must be performed combinatorially with other methods to accurately determine the causes of changes in red or green fluorescence levels. Moreover, MitoTimer is a very useful tool to indirectly quantify mitophagy [70]. This assay quantifies several pathophysiological parameters of mitochondria, accurately assesses their health status, and plays potentially important roles in future studies [62, 71, 72]. Moreover, mitoKeima is also a fluorophore that can help quantify mitophagy. Changes in the emission wavelength of mitoKeima depend on the acidity (red color) or neutrality (green color) of the pH of the microenvironment, e.g., during the transfer from an autophagosome (green color) to a lysosome (red color). Hence, mitoKeima is a powerful pH-dependent fluorophore that is more specific for monitoring mitophagy than MitoTimer [73, 74].

#### Determination of mitochondrial mass

Mitophagy is accompanied by a reduction in mitochondrial mass; therefore, direct detection of changes in mitochondrial mass helps detect mitophagy. Mitochondria are labeled with mitochondrial dyes or corresponding antibodies, and the number of mitochondria per cell is determined via flow cytometry. RT-PCR may be used to quantify mtDNA [75-77]. These methods directly quantify mitochondrial mass. Simultaneously, mitochondrial mass is regulated via numerous regulatory mechanisms, and mitochondrial biogenesis and degradation are reportedly two of the most prominent regulatory mechanisms. Therefore, the decline in mitochondrial mass may not only be affected by mitophagy but also a process involving multiple regulatory mechanisms. Hence, determination of mitochondrial mass is an adjunct method of detecting mitophagy [78].

### 2.3 Factors inducing mitophagy

During cell growth and development, numerous endogenous or exogenous stimulants can alter the degree of mitophagy. Therefore, mitophagy is involved in various pathophysiological processes.

#### Mitophagy and stress

In the cardiovascular system, mitophagy exerts protective effects in response to cellular starvation. Mitophagy is activated during starvation to regulate mitochondrial quality and quantity through elimination of damaged or redundant mitochondria. Mitophagy is enhanced during cellular hypoxia. Acetylcholine reduces the damage caused by hypoxia/reoxygenation by increasing mitophagy in H9C2 cells. Violent exercise alters mitophagy levels by adaptively regulating mitochondrial function [79-81].

#### Mitophagy and the cellular defense

During intracellular pathogenic infections, mitophagy is induced to defend against pathogens. Pathogens also induce mitophagy and disrupt mitochondrial homeostasis. For example, sepsis induces reversible cardiac mitochondrial injury, and recovery is reportedly associated with mitophagy based on morphological and biochemical evidence, suggesting that mitophagy is activated and plays an important role during cardiac recovery from sepsis [82, 83].

#### Mitophagy and the maintenance of cellular homeostasis

In normal cells, mitophagy may have a housekeeping function and renew mitochondrial components. Mitophagy participates in metabolic remodeling and is closely associated with inflammatory response factors. For example, orally administered polyaminospermine in mice enhances mitophagy, inhibits subclinical inflammation, and increases longevity. Changes in the degree of mitophagy modulate stem cell activity and chronic cardiomyocyte inflammation [19, 84].

#### Mitophagy and the regulation of cell growth and development

Mitophagy alters the oxidative respiratory chain activity by regulating mitochondrial quantity and quality, thus contributing to the regulation of cell growth and development. For example, macrophage fibrosis factor (Mff)-deficient mice died of dilated cardiomyopathy at 13 weeks. Mitophagy was significantly elevated in their mutated tissues [84, 85].

#### Mitophagy and aging

Cellular aging has a prominent mitochondrial basis. Mitophagy reportedly mediates various degenerative diseases and contributes to their pathophysiology and treatment. For example, mitophagy is disrupted in the heart of aged individuals and in senescent cells and DOX-treated hearts and cells [19, 86].

## 3. Mitophagy and cardiovascular disease

Since mitophagy is involved in numerous prominent pathophysiological phenomena, it contributes to CVD pathogenesis.

### 3.1 Ischemic heart disease

Ischemic heart disease refers to myocardial damage caused by an imbalance between coronary blood flow and myocardial demand. Ischemic heart disease is a serious threat to human health. Ischemia causes myocardial cell injury and death [29]. Modern therapies can restore coronary circulation and achieve reperfusion, thus potentially reducing cardiac damage. Nevertheless, the recovery of blood supply has an irreversible adverse effect known as ischemia/reperfusion (I/R) injury [87, 88]. As the energy-supplying organelles in cardiomyocytes, mitochondria are major targets of I/R injury. I/R induces the loss of mitochondrial cristae, reduces mitochondrial membrane potential, and opens the mitochondrial permeability transition pore (mPTP) [89]. These effects lead to mitochondrial damage and an aggravation of the imbalance of mitophagy (excessive inhibition or promotion) [28, 89, 90]. Mitophagy protects myocardial cells during I/R injury. I/R reduces mitophagy and stimulates apoptosis in cardiomyocytes [91]. An appropriate increase in mitophagy potentially mitigates I/R injury-induced cardiomyocyte apoptosis. Simultaneously, however, mitophagy may also play a negative role in I/R injury. Inhibition of mitophagy potentially protects the myocardium against I/R injury, reduce cardiomyocyte apoptosis, improve cardiac function, and protect mitochondrial integrity[15, 92, 93]. Key mitochondrial markers PINK1 and Parkin were reportedly upregulated and enhanced Parkin transfer and activation during I/R injury [94]. I/R injury promotes BNIP3 upregulation and FUNDC1 downregulation [95, 96]. LC3 affinity for BNIP3 and FUNDC1 depends upon its phosphorylation levels. I/R injury potentially increases BNIP3 phosphorylation at S17, enhance the binding affinity of LC3, and elevate mitophagy. During I/R, phosphorylation at Y18 and S13 are increased, LC3 affinity decreased with a reduction in mitophagy [97-99].

### 3.2 Diabetic cardiomyopathy

Diabetic cardiomyopathy (DCM) is a major manifestation of organ damage in diabetic complications. DCM is an important cause of morbidity and mortality in individuals with diabetes. Mitochondrial dysfunction is a prominent contributor to diabetic cardiomyopathy [100]. Mitochondria are the primary sources of ROS and targets for oxidative stress injury. In the diabetic myocardium, damaged mitochondria produce large amounts of ROS, which exacerbates mitochondrial damage and triggers a vicious cycle of cardiomyocyte death [101-104]. In individuals with diabetic cardiomyopathy and in animal and cellular models, impaired mitochondrial structure and function and increased ROS production have been commonly observed. In vivo analyses using animals revealed that antioxidant therapy protects against diabetic cardiomyopathy [105]. However, clinical antioxidant therapy did not exert the same effect possibly because of the sustained release of ROS from damaged mitochondria [106]. Blocking of continuous ROS synthesis via mitophagy is a potential key strategy to treat diabetic cardiomyopathy [102, 106-108]. Nevertheless, the functional status and regulatory mechanisms of mitophagy in the diabetic myocardium are unknown. Mitophagy levels in a diabetic cardiomyopathy model were significantly different from those in the control. While cardiac mitophagy was inhibited in a type 1 diabetic mouse model, it was actually promoted in a type 2 diabetic mouse model [109-113]. It is yet unclear whether mitophagy protects against or induces injury in the pathogenesis of diabetic cardiomyopathy. Relative to the control, the contents of mitophagy-related proteins were significantly altered in several diabetic tissues including the heart. In male prediabetic rats, the generation of mitochondrial ROS increased and BNIP3 was downregulated in comparison with the corresponding values in the controls [14, 114]. In types 1 and 2 diabetes models, PINK1 and Parkin were downregulated [109, 115, 116]. In the vascular smooth muscle cells of diabetic mice, PINK1 and Parkin were upregulated and mitophagy was induced relative to the control [117].

### 3.3 Heart failure

Heart failure (HF) is characterized by high morbidity and mortality and is the terminal stage in various heart diseases [118, 119]. Current methods of treating heart failure may mitigate or alleviate symptoms; however, the prognosis remains poor. To better determine treatment methods for heart failure, it is necessary to understand oxidative stress and chronic inflammation associated with this condition [119-121]. During heart failure, mitochondrial dysfunction is a common pathophysiological phenomenon, which has received increasing attention. Mitophagy is decreased with aging or disease [122]. Consequently, elimination of damaged mitochondria is inadequate, ROS and peroxide levels are increased. Cardiomyocyte mitochondrial proteins, lipids, and DNA undergo oxidative damage and contribute to heart failure. These findings corroborate the clinical presentation of heart failure in aged patients and those with terminal heart disease [17, 26, 123]. Biopsies of individuals with heart failure have revealed that the autophagy-specific genes beclin1 and LC3-II were downregulated after treatment [124]. Therefore, we predicted that mitophagy is associated with the occurrence and development of heart failure. Mfn2 regulates heart failure-related mitophagy by altering the mitochondrial membrane potential [125]. Subsequent experiments on heart failure models have revealed that insufficient mitophagy aggravates heart injury[122]. PINK1 is generally downregulated in individuals with heart failure, indicating a reduction in mitophagy levels. However, it is uncertain whether PINK1 downregulation is the cause or effect of heart failure [126, 127]. In vivo studies using animals confirmed the role of PINK1 in heart failure. PINK1 knockout mice were more susceptible to overload-induced heart stress and consequent heart failure than wild type mice [128]. Parkin-mediated mitophagy decreases with age [122]. Moreover, the number of dysfunctional mitochondria increases with age until it surpasses the Parkin-mediated scavenging ability of mitophagy. With aging, myocardial mitophagy becomes inadequate to maintain the normal homeostatic of mitochondrial function. In mice, Parkin deficiency has resulted in the accumulation of dysfunctional cardiomyocyte mitochondria, which elevates the risk of heart failure after myocardial infarction [28]. In mouse heart failure models induced via increased stress, Nix-silenced mice presented less myocardial fibrosis and more stable systolic function than wild type mice [129]. Thus far, myocardial mitophagy has not been reportedly increased in individuals with heart failure. Nevertheless, an increase in mitophagy leads to excessive mitochondrial clearance, and the remaining mitochondria cannot accommodate the energy demand of cardiomyocytes. This situation is extremely unfavorable for individuals with heart failure

### 3.4 Hypertension

Hypertension is a systemic disease with a high incidence and is closely associated with cardiovascular, cerebrovascular, kidney, and other diseases [130]. There is no direct evidence for the role of mitophagy in the pathophysiology of hypertension. Complications of hypertension such as atherosclerosis, myocardial I/R injury and hypertrophy, and heart failure are closely associated with cardiomyocyte and endothelial mitochondrial dysfunction [131]. Insufficient energy supply, kinetic imbalance, oxidative damage, abnormal signal transduction, and mitochondrial dysfunction caused by genetic mutations are risk factors for hypertension [132-134]. Myocardial hypertrophy associated with hypertension reportedly altered cardiomyocyte phenotype, function, and energy metabolism. These changes were reflected in the utilization of metabolic substrates and the function of the ETC, ATP synthesis, and other processes [135]. ROS regulate vascular structure and tension remodeling. An increase or retention of ROS may cause vasoconstriction, decrease vascular diastolic function, damage smooth muscle cells, promote vascular inflammation and remodeling, increase peripheral vascular resistance, and aggravate hypertension [136]. Mfn1, Mfn2, and OPA1 (optic atrophy 1) mRNA levels, which are associated with mitochondrial dynamics, were decreased in hypertensive rat heart models. Hence, mitochondrial fragmentation is increased in hypertension [137]. Mitochondrial division is a compensatory mechanism maintaining cardiac contraction in hypertension and exacerbating ventricular wall thickening [138]. Hypertension is suggested to alter mitochondrial function, energy metabolism, and mitochondrial dynamics in cardiomyocytes [131]. Clearance of mitochondrial dysfunction is closely associated with mitophagy. Therefore, elucidation of the role of mitophagy in the occurrence and development of hypertension may be important to investigate the pathophysiology of this condition and novel treatments for this condition.

### 3.5 Atherosclerosis

Atherosclerosis (AS) is a chronic disease characterized by lipid accumulation, vascular smooth muscle cell proliferation, apoptosis, and local inflammation [139]. The pathogenesis of atherosclerosis is complex and includes abnormal lipid metabolism, inflammation, endothelial injury, oxidative stress, and other mechanisms [140, 141]. In the pathophysiology of atherosclerosis, plaque tissue stability determines disease occurrence and progression [142]. In plaque macrophages, vascular smooth muscle cells, and endothelial cells, mitophagy clears the damaged mitochondria, stabilizes the mitochondrial function in the damaged cells, reduces cell damage, maintains plaque integrity, and prevents disease progression caused by plaque tissue rupture [143]. Oxidized low-density lipoprotein (ox-LDL) has been widely used to generate atherosclerosis models because it is an atherosclerotic agent. In vascular smooth muscle cells, ox-LDL disrupts mitochondrial structure, increases ROS levels, and induces mitochondrial dysfunction [144]. Furthermore, Ox-LDL activates mitophagy, which eliminates nonfunctional and damaged mitochondria. After the addition of mitophagy inhibitors, mitophagy and its related pathway proteins are significantly decreased, and cell apoptosis is significantly increased [145]. Mitophagy protects against atherosclerotic stress-induced apoptosis in human vascular smooth muscle cells (VSMCs) [146-148]. PINK1/Parkin was reportedly upregulated in atherosclerotic disease models than in normal tissues. PINK1 overexpression enhances the protective effect of mitophagy on VSMCs, whereas PINK1 gene knockout counteracts it [146, 147]. Hence, mitophagy is a potential target for the stabilization of atherosclerotic plaques.

### 3.6 Arrhythmia

Arrhythmia is a prominent aspect of CVD. It may occur individually or in association with other cardiovascular disease. In a healthy heart, coordinated electrical propagation supports cardiac function; upon failure of such a cardiac action potential, cardiac arrhythmia occurs [149]. Although there is no direct evidence of the role of mitophagy in arrhythmia, extensive evidence indicates that mitochondria play a functional role in arrhythmia. Mitochondria are involved in arrhythmia owing to their ability to produce ATP and ROS [150]. Mitochondrial dysfunction reduces ATP production, affects cardiac electrical conduction, thus altering sarcolemmal K^+^ fluxes via ATP-sensitive potassium channels [151]. However, excessive mitochondrial ROS production can introduce heterogeneity in cardiac action potentials [152]. Alterations in ATP and ROS levels are based on a vicious cycle: during mitochondrial dysfunction, the mitochondrial membrane potential reduced, thus decreasing ATP and increasing ROS levels; thereafter, further mitochondrial dysfunction is induced, with the mitochondrial membrane potential reduced, with a concomitant reduction in ATP generation. This vicious cycle causes electrophysiological alterations, thereby causing arrhythmia [150, 153]. Proper mitophagy can eliminate damaged mitochondria, maintain mitochondrial homeostasis in cells, and prevent damage to hazardous substances, thus further disrupting cellular phenomena. During arrhythmia, changes in ATP and ROS levels caused by mitochondrial damage play an important role in mitophagy. Therefore, we can boldly speculate that mitophagy eliminates damaged mitochondria and regulates ROS and ATP levels to a certain extent, thereby inhibiting or decelerating the occurrence of arrhythmia.

### 3.7 Stroke

Stroke is an acute cerebrovascular disease, which can be categorized as ischemic stroke and hemorrhagic stroke in accordance with its pathogenesis. Ischemic stroke is caused by atherosclerotic plaque disruption and spontaneous intracranial hemorrhage and is often caused by hypertension [154, 155]. Current studies on stroke have revealed significant advancements; however, current treatments still have risks [156]. In ischemic stroke, mitochondrial aggregation events that cause cell death and tissue infarction primarily result from a lack of nutrients and oxygen and reduction in ATP, and during the reperfusion phase, extensive cellular damage are caused by a mitochondrial Ca^2+^ overload, mPTP opening, and excessive production of ROS [157, 158]. In hemorrhagic stroke, mitochondrial dysfunction decreases mitochondrial respiratory function, and ischemia decreases oxygen metabolism in hematoma [159]. Hence, the regulation of mitochondrial homeostasis is an apparently promising therapy for stroke, and mitophagy serves as an important underlying mechanism. However, mitophagy is a double-edged sword in stroke; there is no consensus regarding whether mitophagy plays a protective or damaging role in stroke. In in vivo and in vitro models of stroke, BNIP3 and Nix are reportedly upregulated, leading to delayed neuronal death [160]; however, subsequent studies reported that Nix-mediated mitophagy protects against ischemic stroke [161]. PINK1/Parkin-mediated mitophagy is activated in brain damage induced by ischemia stroke; however, the underlying function is unclear [162]. Although the function of mitophagy is still unclear in stroke, it is generally accepted that the degree of mitophagy is the determinant during stroke, and physiological levels can be beneficial, while excessive or inadequate levels could be deleterious [30, 163].

**Table1:** Therapeutic application of mitophagy.

Diseases	Representatives	Mechanisms	Effects	References
MicroRNAs				
I/R	MiR-410	Mitophagy-	damage	[165]
I/R	MiR-137	BNIP3/FUNDC1-	-	[166]
Cardiac Lipotoxicity, DCM	MiR-133a	Nix-	protection	[167]

Clinical drugs and chemical reagents				

I/R	Melatonin	PINK1/Parkin-	protection	[168]
DCM	Melatonin	PINK1/Parkin+	protection	[169]
AS	Melatonin	PINK1/Parkin+	protection	[170]
I/R	Simvastatin	Parkin/P62+	protection	[171]
I/R	Liraglutide	Parkin+	protection	[172]
I/R	Zine	PINK1+	protection	[173]
I/R	Sevoflurane postconditioning	Parkin-	protection	[174]
I/R	TEMPOL preconditioning	PINK1/Parkin+	protection	[175]
HF	Curcumin	BNIP3-	protection	[176]
Cardiotoxicity	Ellagic acid	BNIP3-	protection	[177]
Stroke	Tunicamycin and thapsigargin	Mitophagy+	protection	[178]
Stroke	Peroxynitrite	PINK1/Parkin+	damage	[179]
Stroke	Naringin	Parkin-	protection	[180]

Signal pathways				

AS	NR4A1/CaMKII activation	Parkin+	damage	[145]
Stroke	MAPK-ERK-CREB blockade	Mfn2-	damage	[181]
I/R	Rab5 endosomal pathway activation	Parkin+	protection	[182].
I/R	P53/TIGAR activation	BNIP3-	damage	[183]
HF	JNK/FOXO3a activation	BNIP3+	damage	[184].

Activators/inhibitors, genes knock in/out				

I/R	STAT1 activation	Mitophagy-	damage	[185]
AS	PINK1/Parkin knockout	PINK1/Parkin-	damage	[146, 147]
I/R	GPER activation	PINK1/Parkin-	protection	[93]
I/R	ALDH2 activation	PINK1/Parkin-	protection	[186]
DCM	Sirt3 overexpression	Parkin+	protection	[187]
DCM	Mst1 knockout	Parkin+	protection	[188]
HF	BAG3 knockdown	Parkin-	damage	[189]
AS	F13A	PINK1/Parkin-	protection	[190]
HF	CsA	PINK1/Parkin-	protection	[191]
HF	Akt2 knockout	BNIP3/PINK1/Parkin+	protection	[192]
Stroke	Nix knockout	Nix-	damage	[161]
I/R	DUSP1 activation	BNIP3-	protection	[98]
HF	SWI/SNF deletion	BNIP3+	damage	[193]
I/R	FUNDC1 knockout	FUNDC1-	damage	[96]

Environmental stimuli				

I/R	Mild hypothermia	Parkin-	protection	[194]
I/R	Hypoxic preconditioning	FUNDC1+	protection	[96]
Myocardial inflammatory	Acute exercise	BNIP3+	protection	[80]
I/R	Exercise preconditioning	Parkin+	protection	[195]
Stroke	Acidic postconditioning	Mitophagy+	protection	[196]
Stroke	Remote ischemic post conditioning	Parkin+	protection	[197]

## 4. Therapeutic application of mitophagy

Mitophagy plays an important role in CVD pathogenesis and is, therefore, a potential therapeutic target. CVD can be treated, or its progress can be delayed by promoting or inhibiting mitophagy, thus maintaining the functional stability of mitochondria and reducing cell damage in the disease state (Table 1).

### 4.1 MicroRNAs

MicroRNAs are small, single-stranded, noncoding RNA molecules which inhibit the translation of target mRNAs or induce their degradation [164]. MiR-410 is reportedly significantly upregulated in an I/R injury model of human adult cardiomyocytes (HACMs), which presented decreased mitochondrial function and loss of mitophagy. MiR-410 overexpression reportedly decreased cell viability, ATP generation, mitochondrial membrane potential, and mitophagy. In contrast, miR-410 downregulation had the opposite effect. MiR-410 directly targeted HSPB1 and regulated its activity and mitophagy [165]. MiR-137 is reportedly significantly upregulated under hypoxia, wherein it downregulates Nix and FUNDC1 and impairs mitophagy [166]. In lipid toxicity and diabetic mouse models, miR-133a was reportedly downregulated and Nix was upregulated. MiR-133a upregulation inhibited Nix translation, regulated mitochondrial function, and stabilized mitochondrial membrane potential [167].

### 4.2 Clinical drugs and chemical reagents

Certain drugs and chemicals regulate mitophagy. Melatonin prevents the opening of the mPTP and the activation of PINK1/Parkin in the microcirculating endothelial cells of an I/R mouse model. Melatonin prevents mitophagy-mediated cell death and protects cardiac microvessels from I/R injury; the underlying mechanism may be attributed to the inhibitory effects of melatonin on mitochondrial fission VDAC1 HK2 mPTP mitophagy axis via activation of AMPKα [168]. Furthermore, melatonin restores mitophagy in diabetic cardiomyopathy probably via Parkin translocation and Mst1 repression [169]. Subsequent studies reported that melatonin induced mitophagy through the Sirt3/FOXO3a/Parkin signaling pathway, attenuated NLRP3 (NLR family pyrin domain containing 3) inflammasome activation, and prevented the progression of atherosclerosis [170]. Simvastatin suppresses mTOR signaling, triggers mitochondrial translocation of Parkin and p62/SQSTM1, activates mitophagy, and reduces the infarct area in mouse and HL-1 cell models of myocardial infarction. Coenzyme Q may influence the anti-ischemic effect of statins by interfering with mitophagy [171]. Liraglutide upregulated SIRT1 and Parkin, activated mitophagy, reduced cellular oxidative stress, balanced the redox reaction, and maintained mitochondrial homeostasis. These effects promoted myocardial repair after myocardial infarction [172]. In both healthy and I/R rat cardiomyocytes, zinc (Zn) upregulated PINK1 and Beclin1, activated mitophagy, inhibited the generation of superoxide mitochondria, reduced the loss of mitochondrial membrane potential during reperfusion, prevented mitochondrial oxidative stress, and protected heart damage [173]. Sevoflurane posttreatment downregulated Parkin in a rat I/R model, inhibited mitophagy, maintained ATP, reversed mitochondrial damage, and protected the heart [174]. Pretreatment with the antioxidant TEMPOL upregulated PINK1 and Parkin, restored mitophagy in the aged myocardium upon isoproterenol (ISO) treatment, and promoted cardiac recovery in aging animals [175]. BNIP3 binding activated acetyltransferase p300 and increased histone acetylation and transcription factor GATA4 levels. Curcumin inhibited these phenomena and reduced mitophagy levels [176]. Erythorbic acid (EA) reduced mitochondrial injury and necrotic cell death of cardiac myocytes and alleviated oxidative damage and cardiac dysfunction during anthracycline therapy by functionally abrogating Bnip3 activity, thus inhibiting BNIP3-induced mitophagy [177]. Tunicamycin and thapsigargin-induced ER stress protects against ischemic stroke injury, which is probably involved in mitophagy induction [178]. Peroxynitrite-induced PINK1/Parkin-mediated mitophagy activation through drp1 recruitment to damaged mitochondria aggravates cerebral I/R injury during stroke [179]. A natural antioxidant naringin potentially inhibits peroxynitrite-mediated mitophagy activation by inhibiting the translocation of Parkin to the mitochondria and attenuating ischemic stroke injury [180].

### 4.3 Signaling pathways

Several signaling pathways regulate molecules that control mitophagy. In a mouse atherosclerosis model established with ox-LDL, NR4A1 was significantly upregulated and controlled Parkin activation through posttranscriptional CaMKII modification. Thus, it activated Parkin-mediated mitophagy. Excessive mitophagy reduced mitochondrial quality which, in turn, caused energy shortage and mitochondrial dysfunction [145]. Furthermore, blockade of the MAPK-ERK-CREB signaling pathway upregulated NR4A1 and repressed Mfn2-mediated mitophagy, thus expanding the area of cerebral infarction and increasing neuronal apoptosis [181]. In cardiomyocytes, damaged mitochondria are sequestered in rab5-positive early endosomes via the ESCRT mechanism, which depends on Parkin, and the loss of rab5 function increases the susceptibility of embryonic fibroblasts and cardiomyocytes to cell death [182]. In a mouse ischemic model, activation of the p53-TIGAR axis downregulated BNIP3, inhibited mitophagy, caused the accumulation of damaged mitochondria, and weakened the cardioprotective effect [183]. The JNK signaling pathway is a key regulator of the FOXO3a transcription factor, which promotes BNIP3 expression in heart failure models. JNK influences the degree of mitophagy by regulating the level of BNIP3[184].

### 4.4 Activators/inhibitors and gene knock in/out

Certain receptor antagonists, inhibitors of upstream molecules, and gene knockouts directly or indirectly regulate mitophagy. STAT1 is not only localized in the mitochondria but also serves as an LC3b binding partner; hence, STAT1 decreases mitophagy and promotes cell death in the myocardial I/R response [185]. Silencing of PINK1 and Parkin reduced mitophagy in atherosclerosis and enhanced apoptosis in VSMCs via ox-LDL. In contrast, PINK1 and Parkin overexpression inhibited cell death [146, 147]. G protein-coupled estrogen receptor 1 (GPER) agonist increases GPER activity, reduces Parkin translocation from the cytosol to the mitochondria, and downregulates PINK1 protein expression, inhibiting the PINK1/Parkin pathway and mitophagy, protecting mitochondrial structural integrity and function, and protecting the heart after I/R injury [93]. Both in I/R rats and hypoxia/reoxygenation H9C2 cells, ALDH2 activation suppressed phosphatase and PINK1/Parkin expression, preventing 4-hydroxynonenal, ROS, and mitochondrial superoxide accumulation, and regulating mitophagy, thus protecting the heart against I/R injury [186]. In a mouse model of diabetic cardiomyopathy, Sirt3 overexpression activated deacetylation of Foxo3A and expression of Parkin, upregulated Parkin-dependent mitophagy, inhibited mitochondrial damage and apoptosis in cardiomyocytes, and played an important role in the occurrence and development of diabetic cardiomyopathy [187]. Mst1 knockout reportedly significantly upregulated Parkin, enhanced mitochondrial translocation, and protected the myocardium of diabetic mice. These effects may be associated with Sirt3 downregulation via Mst1 [188]. BAG3 functions upstream of Parkin, and BAG3 knock-down reduced PINK1/Parkin-mediated mitophagy and impaired the clearance of defective mitochondria, thus increasing levels of toxicity within the cells and subsequent cell death in the context of heart failure [189]. In AS, apelin-13 increases mitophagy by activating the PINK1/Parkin pathway, and F13A is an apelin-13 antagonist in PINK1 and Parkin-dependent mitophagy, which blocks the effect of apelin-13 in the progression of atherosclerosis [190]. Both CsA and PINK1 knockout significantly downregulated PINK1 and Parkin proteins in senescent cardiomyocytes, thus reducing the level of mitophagy and blocking cardiomyocyte senescence [191]. Akt2 knockout prevented cardiac aging by upregulating Foxo1-related BNIP3, PINK1, and Parkin and by maintaining mitochondrial integrity [192]. Nix knockout impaired mitophagy and aggravated ischemic stroke in mice, which can be rescued via Nix overexpression [161]. DUSP1 overexpression inhibited BNIP3 activation, thereby inactivating the JNK pathway and alleviating mitophagy. This mechanism improved the survival of myocardial tissue after I/R[98]. After SWI/SNF was deleted in Brg1/Brm double-mutant mice, BNIP3 was upregulated and mitophagy was increased. Consequently, small and fragmented mitochondria were formed. This reaction corresponded with changes occurring during heart failure[193]. In the I/R model of FUNDC1 knockout mice, mitophagy was inhibited and cardiac injury was aggravated [96].

### 4.5 Environmental stimuli

Certain environmental stimuli can inhibit or promote mitophagy and alter the content of the proteins related to this phenomenon. Mild hypothermia downregulated Parkin in hippocampal neurons in a cardiac arrest model, diminishing mitophagy in the neurons, protecting mitochondria, and improving neurological function after cardiac arrest [194]. Hypoxic preconditioning induced FUNDC1-dependent activation of mitophagy and decreased I/R-induced cardiac injury [96]. Myocardial mitochondrial function adapts to stress during acute exercise and manifests as significant upregulation of the mitophagy-related protein BNIP3, which stimulates mitophagy and minimizes myocardial injury [80]. Simultaneously, exercise preconditioning significantly suppresses exhaustive exercise-induced hypoxia-ischemia injuries through upregulated parkin-dependent mitophagy [195]. Acidic postconditioning-induced mitophagy renders the brain resistant to ischemic stroke through recruitment of PARK2 to mitochondria [196]. Remote ischemic post-conditioning promoted mitophagy via Parkin upregulation and inhibited oxidative stress responses, thus mitigating cerebral I/R injury in a rat model of stroke [197].

**Figure 2.**
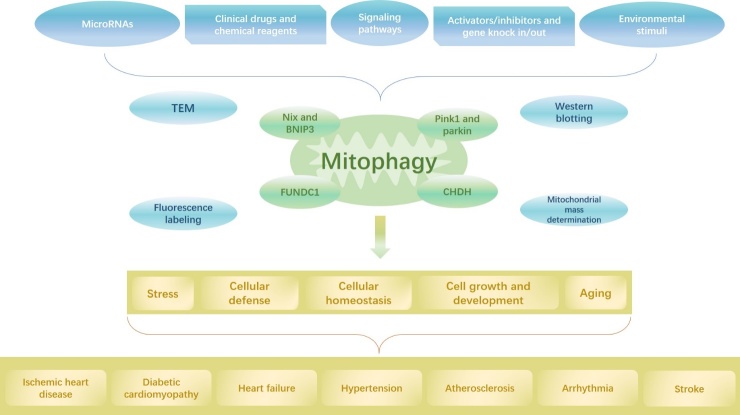
The graphical abstract. Mitophagy plays an important role in cardiovascular disease, and the degree of mitophagy can be detected via TEM, western blotting, fluorescence labeling, and mitochondrial mass determination, and the related molecular mechanism depends on PINK1/Parkin, CHDH, Nix/BNIP3, FUNDC1, etc. Mitophagy is related with certain physiological and pathological phenomena including stress, cellular defense, maintenance of cellular homeostasis, regulation of cell growth and development, and aging; these phenomena are also involved in the pathogenesis of cardiovascular diseases including ischemic heart disease, diabetic cardiomyopathy, heart failure, hypertension, atherosclerosis, arrhythmia, and stroke, and these diseases are closely associated with mitophagy. Therefore, certain factors including microRNAs, clinical drugs and chemical reagents, signaling pathways, activators/inhibitors and gene knock in/out, and environmental stimuli can regulate the level of mitophagy to alter the progression of these diseases.

## 5. Conclusion and Outlook

Mitochondrial injury and dysfunction are prominent pathophysiological mechanisms of CVD. Mitophagy potentially mitigates or alleviates these disorders and maintains cellular stability. The underlying mechanisms are complex, interrelated, and regulated by numerous stimulating factors. Several factors induce mitophagy during cellular growth, development, aging, and death. Alterations in the degree of mitophagy may alter the course of cardiovascular disease. Therefore, mitophagy is a potential therapeutic target for cardiovascular disease (Fig. 2). Nevertheless, it is yet unclear whether mitophagy retards or accelerates damage in CVD pathogenesis. Furthermore, it is unclear whether mitophagy is mediated by the unique functions of different molecules. Further studies are required to investigate whether the mechanisms underlying mitophagy induced by various molecules have mutual effects and the role of mitophagy in CVD, thus leading to advancements in the development of clinical diagnoses and treatments.

## References

[1] Joseph P, Leong D, McKee M, Anand SS, Schwalm JD, Teo K, et al (2017). Reducing the Global Burden of Cardiovascular Disease, Part 1: The Epidemiology and Risk Factors. Circ Res, 121:677-694. 28860318 10.1161/CIRCRESAHA.117.308903

[2] Dehghan M, Mente A, Zhang X, Swaminathan S, Li W, Mohan V, et al (2017). Associations of fats and carbohydrate intake with cardiovascular disease and mortality in 18 countries from five continents (PURE): a prospective cohort study. Lancet, 390:2050-2062. 28864332 10.1016/S0140-6736(17)32252-3

[3] Naghavi M, Abajobir A, Abbafati C, Abbas K, Abd-Allah F, Abera S, et al (2017). Global, regional, and national age-sex specific mortality for 264 causes of death, 1980-2016: a systematic analysis for the Global Burden of Disease Study 2016. Lancet, 390:1151-1210. 28919116 10.1016/S0140-6736(17)32152-9 PMC5605883

[4] Yusuf S, Rangarajan S, Teo K, Islam S, Li W, Liu L, et al (2014). Cardiovascular risk and events in 17 low-, middle-, and high-income countries. N Engl J Med, 371:818-827. 25162888 10.1056/NEJMoa1311890

[5] Bonora M, Wieckowski MR, Sinclair DA, Kroemer G, Pinton P, Galluzzi L (2019). Targeting mitochondria for cardiovascular disorders: therapeutic potential and obstacles. Nat Rev Cardiol, 16:33-55. 30177752 10.1038/s41569-018-0074-0 PMC6349394

[6] Galluzzi L, Kepp O, Trojel-Hansen C, Kroemer G (2012). Mitochondrial control of cellular life, stress, and death. Circ Res, 111:1198-1207. 23065343 10.1161/CIRCRESAHA.112.268946

[7] Lopez-Crisosto C, Pennanen C, Vasquez-Trincado C, Morales PE, Bravo-Sagua R, Quest AFG, et al (2017). Sarcoplasmic reticulum-mitochondria communication in cardiovascular pathophysiology. Nat Rev Cardiol, 14:342-360. 28275246 10.1038/nrcardio.2017.23

[8] Nah J, Miyamoto S, Sadoshima J (2017). Mitophagy as a Protective Mechanism against Myocardial Stress. Compr Physiol, 7:1407-1424. 28915329 10.1002/cphy.c170005

[9] Shirakabe A, Ikeda Y, Sciarretta S, Zablocki DK, Sadoshima J (2016). Aging and Autophagy in the Heart. Circ Res, 118:1563-1576. 27174950 10.1161/CIRCRESAHA.116.307474 PMC4869999

[10] Nah J, Fernandez AF, Kitsis RN, Levine B, Sadoshima J (2016). Does Autophagy Mediate Cardiac Myocyte Death During Stress? Circ Res, 119:893-895. 27688304 10.1161/CIRCRESAHA.116.309765 PMC5161244

[11] Lemasters JJ (2005). Selective mitochondrial autophagy, or mitophagy, as a targeted defense against oxidative stress, mitochondrial dysfunction, and aging. Rejuvenation Res, 8:3-5. 15798367 10.1089/rej.2005.8.3

[12] Dorn GW 2nd, Vega RB, Kelly DP (2015). Mitochondrial biogenesis and dynamics in the developing and diseased heart. Genes Dev, 29:1981-1991. 26443844 10.1101/gad.269894.115 PMC4604339

[13] Lesnefsky EJ, Chen Q, Hoppel CL (2016). Mitochondrial Metabolism in Aging Heart. Circ Res, 118:1593-1611. 27174952 10.1161/CIRCRESAHA.116.307505 PMC5009371

[14] Liang Q, Kobayashi S (2016). Mitochondrial quality control in the diabetic heart. J Mol Cell Cardiol, 95:57-69. 26739215 10.1016/j.yjmcc.2015.12.025 PMC6263145

[15] Moyzis AG, Sadoshima J, Gustafsson AB (2015). Mending a broken heart: the role of mitophagy in cardioprotection. Am J Physiol Heart Circ Physiol, 308:H183-192. 25437922 10.1152/ajpheart.00708.2014 PMC4312945

[16] Saito T, Sadoshima J (2015). Molecular mechanisms of mitochondrial autophagy/mitophagy in the heart. Circ Res, 116:1477-1490. 25858070 10.1161/CIRCRESAHA.116.303790 PMC4419704

[17] Shi R, Guberman M, Kirshenbaum LA (2018). Mitochondrial quality control: The role of mitophagy in aging. Trends Cardiovasc Med, 28:246-260. 29287956 10.1016/j.tcm.2017.11.008

[18] Shirihai OS, Song M, Dorn GW 2nd, (2015). How mitochondrial dynamism orchestrates mitophagy. Circ Res, 116:1835-1849. 25999423 10.1161/CIRCRESAHA.116.306374 PMC4443843

[19] Sun N, Youle RJ, Finkel T (2016). The Mitochondrial Basis of Aging. Mol Cell, 61:654-666. 26942670 10.1016/j.molcel.2016.01.028 PMC4779179

[20] Tong M, Sadoshima J (2016). Mitochondrial autophagy in cardiomyopathy. Curr Opin Genet Dev, 38:8-15. 27003723 10.1016/j.gde.2016.02.006 PMC5028232

[21] Woodall BP, Gustafsson AB (2018). Autophagy-A key pathway for cardiac health and longevity. Acta Physiol (Oxf), 223:e13074. 29660243 10.1111/apha.13074 PMC6527442

[22] Wu MY, Yiang GT, Liao WT, Tsai AP, Cheng YL, Cheng PW, et al (2018). Current Mechanistic Concepts in Ischemia and Reperfusion Injury. Cell Physiol Biochem, 46:1650-1667. 29694958 10.1159/000489241

[23] Baines CP (2010). The cardiac mitochondrion: nexus of stress. Annu Rev Physiol, 72:61-80. 20148667 10.1146/annurev-physiol-021909-135929

[24] Dai DF, Chen T, Wanagat J, Laflamme M, Marcinek DJ, Emond MJ, et al (2010). Age-dependent cardiomyopathy in mitochondrial mutator mice is attenuated by overexpression of catalase targeted to mitochondria. Aging Cell, 9:536-544. 20456298 10.1111/j.1474-9726.2010.00581.x PMC3265170

[25] Kujoth GC, Hiona A, Pugh TD, Someya S, Panzer K, Wohlgemuth SE, et al (2005). Mitochondrial DNA mutations, oxidative stress, and apoptosis in mammalian aging. Science, 309:481-484. 16020738 10.1126/science.1112125

[26] Dai DF, Rabinovitch PS, Ungvari Z (2012). Mitochondria and cardiovascular aging. Circ Res, 110:1109-1124. 22499901 10.1161/CIRCRESAHA.111.246140 PMC3867977

[27] Wallace DC (2005). A mitochondrial paradigm of metabolic and degenerative diseases, aging, and cancer: a dawn for evolutionary medicine. Annu Rev Genet, 39:359-407. 16285865 10.1146/annurev.genet.39.110304.095751 PMC2821041

[28] Kubli DA, Zhang X, Lee Y, Hanna RA, Quinsay MN, Nguyen CK, et al (2013). Parkin protein deficiency exacerbates cardiac injury and reduces survival following myocardial infarction. J Biol Chem, 288:915-926. 23152496 10.1074/jbc.M112.411363 PMC3543040

[29] Anzell AR, Maizy R, Przyklenk K, Sanderson TH (2018). Mitochondrial Quality Control and Disease: Insights into Ischemia-Reperfusion Injury. Mol Neurobiol, 55:2547-2564. 28401475 10.1007/s12035-017-0503-9 PMC5636654

[30] Tang YC, Tian HX, Yi T, Chen HB (2016). The critical roles of mitophagy in cerebral ischemia. Protein Cell, 7:699-713. 27554669 10.1007/s13238-016-0307-0 PMC5055489

[31] Cuervo AM, Wong E (2014). Chaperone-mediated autophagy: roles in disease and aging. Cell Res, 24:92-104. 24281265 10.1038/cr.2013.153 PMC3879702

[32] Farre JC, Krick R, Subramani S, Thumm M (2009). Turnover of organelles by autophagy in yeast. Curr Opin Cell Biol, 21:522-530. 19515549 10.1016/j.ceb.2009.04.015 PMC2725217

[33] Hamacher-Brady A, Brady NR (2016). Mitophagy programs: mechanisms and physiological implications of mitochondrial targeting by autophagy. Cell Mol Life Sci, 73:775-795. 26611876 10.1007/s00018-015-2087-8 PMC4735260

[34] Tan S, Wong E (2017). Mitophagy Transcriptome: Mechanistic Insights into Polyphenol-Mediated Mitophagy. Oxid Med Cell Longev, 2017:9028435. 10.1155/2017/9028435 PMC5463118 28626500

[35] Okamoto K, Kondo-Okamoto N, Ohsumi Y (2009). A landmark protein essential for mitophagy: Atg32 recruits the autophagic machinery to mitochondria. Autophagy, 5:1203-1205. 19770589 10.4161/auto.5.8.9830

[36] Kissova I, Deffieu M, Manon S, Camougrand N (2004). Uth1p is involved in the autophagic degradation of mitochondria. J Biol Chem, 279:39068-39074. 15247238 10.1074/jbc.M406960200

[37] Kanki T, Wang K, Cao Y, Baba M, Klionsky DJ (2009). Atg32 is a mitochondrial protein that confers selectivity during mitophagy. Dev Cell, 17:98-109. 19619495 10.1016/j.devcel.2009.06.014 PMC2746076

[38] Dunn WA Jr, (1990). Studies on the mechanisms of autophagy: formation of the autophagic vacuole. J Cell Biol, 110:1923-1933. 2351689 10.1083/jcb.110.6.1923 PMC2116114

[39] Gustafsson AB, Gottlieb RA (2008). Recycle or die: the role of autophagy in cardioprotection. J Mol Cell Cardiol, 44:654-661. 18353358 10.1016/j.yjmcc.2008.01.010 PMC2423346

[40] Lemasters JJ (2014). Variants of mitochondrial autophagy: Types 1 and 2 mitophagy and micromitophagy (Type 3). Redox Biol, 2:749-754. 25009776 10.1016/j.redox.2014.06.004 PMC4085350

[41] Kabeya Y, Mizushima N, Ueno T, Yamamoto A, Kirisako T, Noda T, et al (2000). LC3, a mammalian homologue of yeast Apg8p, is localized in autophagosome membranes after processing. Embo j, 19:5720-5728. 11060023 10.1093/emboj/19.21.5720 PMC305793

[42] Yoo SM, Jung YK (2018). A Molecular Approach to Mitophagy and Mitochondrial Dynamics. Mol Cells, 41:18-26. 29370689 10.14348/molcells.2018.2277 PMC5792708

[43] Youle RJ, Narendra DP (2011). Mechanisms of mitophagy. Nat Rev Mol Cell Biol, 12:9-14. 21179058 10.1038/nrm3028 PMC4780047

[44] Jin SM, Lazarou M, Wang C, Kane LA, Narendra DP, Youle RJ (2010). Mitochondrial membrane potential regulates PINK1 import and proteolytic destabilization by PARL. J Cell Biol, 191:933-942. 21115803 10.1083/jcb.201008084 PMC2995166

[45] Narendra DP, Jin SM, Tanaka A, Suen DF, Gautier CA, Shen J, et al (2010). PINK1 is selectively stabilized on impaired mitochondria to activate Parkin. PLoS Biol, 8:e1000298. 20126261 10.1371/journal.pbio.1000298 PMC2811155

[46] Greene AW, Grenier K, Aguileta MA, Muise S, Farazifard R, Haque ME, et al (2012). Mitochondrial processing peptidase regulates PINK1 processing, import and Parkin recruitment. EMBO Rep, 13:378-385. 22354088 10.1038/embor.2012.14 PMC3321149

[47] Chaugule VK, Burchell L, Barber KR, Sidhu A, Leslie SJ, Shaw GS, et al (2011). Autoregulation of Parkin activity through its ubiquitin-like domain. Embo j, 30:2853-2867. 21694720 10.1038/emboj.2011.204 PMC3160258

[48] Lazarou M, Sliter DA, Kane LA, Sarraf SA, Wang C, Burman JL, et al (2015). The ubiquitin kinase PINK1 recruits autophagy receptors to induce mitophagy. Nature, 524:309-314. 26266977 10.1038/nature14893 PMC5018156

[49] Chan NC, Salazar AM, Pham AH, Sweredoski MJ, Kolawa NJ, Graham RL, et al (2011). Broad activation of the ubiquitin-proteasome system by Parkin is critical for mitophagy. Hum Mol Genet, 20:1726-1737. 21296869 10.1093/hmg/ddr048 PMC3071670

[50] Park S, Choi SG, Yoo SM, Son JH, Jung YK (2014). Choline dehydrogenase interacts with SQSTM1/p62 to recruit LC3 and stimulate mitophagy. Autophagy, 10:1906-1920. 25483962 10.4161/auto.32177 PMC4502719

[51] Schwarten M, Mohrluder J, Ma P, Stoldt M, Thielmann Y, Stangler T, et al (2009). Nix directly binds to GABARAP: a possible crosstalk between apoptosis and autophagy. Autophagy, 5:690-698. 19363302 10.4161/auto.5.5.8494

[52] Novak I, Kirkin V, McEwan DG, Zhang J, Wild P, Rozenknop A, et al (2010). Nix is a selective autophagy receptor for mitochondrial clearance. EMBO Rep, 11:45-51. 20010802 10.1038/embor.2009.256 PMC2816619

[53] Hanna RA, Quinsay MN, Orogo AM, Giang K, Rikka S, Gustafsson AB (2012). Microtubule-associated protein 1 light chain 3 (LC3) interacts with Bnip3 protein to selectively remove endoplasmic reticulum and mitochondria via autophagy. J Biol Chem, 287:19094-19104. 22505714 10.1074/jbc.M111.322933 PMC3365942

[54] Sandoval H, Thiagarajan P, Dasgupta SK, Schumacher A, Prchal JT, Chen M, et al (2008). Essential role for Nix in autophagic maturation of erythroid cells. Nature, 454:232-235. 18454133 10.1038/nature07006 PMC2570948

[55] Liu L, Feng D, Chen G, Chen M, Zheng Q, Song P, et al (2012). Mitochondrial outer-membrane protein FUNDC1 mediates hypoxia-induced mitophagy in mammalian cells. Nat Cell Biol, 14:177-185. 22267086 10.1038/ncb2422

[56] Wu W, Tian W, Hu Z, Chen G, Huang L, Li W, et al (2014). ULK1 translocates to mitochondria and phosphorylates FUNDC1 to regulate mitophagy. EMBO Rep, 15:566-575. 24671035 10.1002/embr.201438501 PMC4210082

[57] Boudreau DM, Guzauskas GF, Chen E, Lalla D, Tayama D, Fagan SC, et al (2014). Cost-effectiveness of recombinant tissue-type plasminogen activator within 3 hours of acute ischemic stroke: current evidence. Stroke, 45:3032-3039. 25190439 10.1161/STROKEAHA.114.005852

[58] Strappazzon F, Nazio F, Corrado M, Cianfanelli V, Romagnoli A, Fimia GM, et al (2015). AMBRA1 is able to induce mitophagy via LC3 binding, regardless of PARKIN and p62/SQSTM1. Cell Death Differ, 22:419-432. 25215947 10.1038/cdd.2014.139 PMC4326570

[59] Hayashi M, Raimondi A, O'Toole E, Paradise S, Collesi C, Cremona O, et al (2008). Cell- and stimulus-dependent heterogeneity of synaptic vesicle endocytic recycling mechanisms revealed by studies of dynamin 1-null neurons. Proc Natl Acad Sci U S A, 105:2175-2180. 18250322 10.1073/pnas.0712171105 PMC2538894

[60] Hayashi-Nishino M, Fujita N, Noda T, Yamaguchi A, Yoshimori T, Yamamoto A (2009). A subdomain of the endoplasmic reticulum forms a cradle for autophagosome formation. Nat Cell Biol, 11:1433-1437. 19898463 10.1038/ncb1991

[61] Kirkin V, McEwan DG, Novak I, Dikic I (2009). A role for ubiquitin in selective autophagy. Mol Cell, 34:259-269. 19450525 10.1016/j.molcel.2009.04.026

[62] Kim I, Lemasters JJ (2011). Mitochondrial degradation by autophagy (mitophagy) in GFP-LC3 transgenic hepatocytes during nutrient deprivation. Am J Physiol Cell Physiol, 300:C308-317. 21106691 10.1152/ajpcell.00056.2010 PMC3043636

[63] Goiran T, Duplan E, Rouland L, El Manaa W, Lauritzen I, Dunys J, et al (2018). Nuclear p53-mediated repression of autophagy involves PINK1 transcriptional down-regulation. Cell Death Differ, 25:873-884. 29352272 10.1038/s41418-017-0016-0 PMC5943347

[64] Moscat J, Diaz-Meco MT (2009). p62 at the crossroads of autophagy, apoptosis, and cancer. Cell, 137:1001-1004. 19524504 10.1016/j.cell.2009.05.023 PMC3971861

[65] Mathew R, Karp CM, Beaudoin B, Vuong N, Chen G, Chen HY, et al (2009). Autophagy suppresses tumorigenesis through elimination of p62. Cell, 137:1062-1075. 19524509 10.1016/j.cell.2009.03.048 PMC2802318

[66] Nishida Y, Arakawa S, Fujitani K, Yamaguchi H, Mizuta T, Kanaseki T, et al (2009). Discovery of Atg5/Atg7-independent alternative macroautophagy. Nature, 461:654-658. 19794493 10.1038/nature08455

[67] Zhang J, Randall MS, Loyd MR, Dorsey FC, Kundu M, Cleveland JL, et al (2009). Mitochondrial clearance is regulated by Atg7-dependent and -independent mechanisms during reticulocyte maturation. Blood, 114:157-164. 19417210 10.1182/blood-2008-04-151639 PMC2710944

[68] Honda S, Arakawa S, Nishida Y, Yamaguchi H, Ishii E, Shimizu S (2014). Ulk1-mediated Atg5-independent macroautophagy mediates elimination of mitochondria from embryonic reticulocytes. Nat Commun, 5:4004. 24895007 10.1038/ncomms5004

[69] Wilson RJ, Drake JC, Cui D, Zhang M, Perry HM, Kashatus JA, et al (2019). Conditional MitoTimer reporter mice for assessment of mitochondrial structure, oxidative stress, and mitophagy. Mitochondrion, 44:20-26. 29274400 10.1016/j.mito.2017.12.008 PMC6387589

[70] Williams JA, Zhao K, Jin S, Ding WX (2017). New methods for monitoring mitochondrial biogenesis and mitophagy in vitro and in vivo. Exp. Biol. Med. (Maywood), 242:781-787. 28093935 10.1177/1535370216688802 PMC5407538

[71] Ferree AW, Trudeau K, Zik E, Benador IY, Twig G, Gottlieb RA, et al (2013). MitoTimer probe reveals the impact of autophagy, fusion, and motility on subcellular distribution of young and old mitochondrial protein and on relative mitochondrial protein age. Autophagy, 9:1887-1896. 24149000 10.4161/auto.26503 PMC4028338

[72] Hernandez G, Thornton C, Stotland A, Lui D, Sin J, Ramil J, et al (2013). MitoTimer: a novel tool for monitoring mitochondrial turnover. Autophagy, 9:1852-1861. 24128932 10.4161/auto.26501 PMC4028337

[73] Shirakabe A, Fritzky L, Saito T, Zhai P, Miyamoto S, Gustafsson Å B, et al (2016). Evaluating mitochondrial autophagy in the mouse heart. J. Mol. Cell. Cardiol., 92:134-139. 26868976 10.1016/j.yjmcc.2016.02.007

[74] Tong M, Saito T, Zhai P, Oka SI, Mizushima W, Nakamura M, et al (2019). Mitophagy Is Essential for Maintaining Cardiac Function During High Fat Diet-Induced Diabetic Cardiomyopathy. Circ. Res., 124:1360-1371. 30786833 10.1161/CIRCRESAHA.118.314607 PMC6483841

[75] Lagouge M, Argmann C, Gerhart-Hines Z, Meziane H, Lerin C, Daussin F, et al (2006). Resveratrol improves mitochondrial function and protects against metabolic disease by activating SIRT1 and PGC-1alpha. Cell, 127:1109-1122. 17112576 10.1016/j.cell.2006.11.013

[76] Yoshii SR, Kishi C, Ishihara N, Mizushima N (2011). Parkin mediates proteasome-dependent protein degradation and rupture of the outer mitochondrial membrane. J Biol Chem, 286:19630-19640. 21454557 10.1074/jbc.M110.209338 PMC3103342

[77] Chazotte B (2011). Labeling mitochondria with MitoTracker dyes. Cold Spring Harb Protoc, 2011:990-992. 21807856 10.1101/pdb.prot5648

[78] Melser S, Lavie J, Bénard G (2015). Mitochondrial degradation and energy metabolism. Biochim. Biophys. Acta, 1853:2812-2821. 25979837 10.1016/j.bbamcr.2015.05.010

[79] Ikeda Y, Shirakabe A, Maejima Y, Zhai P, Sciarretta S, Toli J, et al (2015). Endogenous Drp1 mediates mitochondrial autophagy and protects the heart against energy stress. Circ Res, 116:264-278. 25332205 10.1161/CIRCRESAHA.116.303356

[80] Li H, Miao W, Ma J, Xv Z, Bo H, Li J, et al (2016). Acute Exercise-Induced Mitochondrial Stress Triggers an Inflammatory Response in the Myocardium via NLRP3 Inflammasome Activation with Mitophagy. Oxid Med Cell Longev, 2016:1987149. 10.1155/2016/1987149 PMC4684864 26770647

[81] Ido MS, Okosun IS, Bayakly R, Clarkson L, Lugtu J, Floyd S, et al (2016). Door to Intravenous Tissue Plasminogen Activator Time and Hospital Length of Stay in Acute Ischemic Stroke Patients, Georgia, 2007-2013. J Stroke Cerebrovasc Dis, 25:866-871. 26853143 10.1016/j.jstrokecerebrovasdis.2015.12.025

[82] Piquereau J, Godin R, Deschenes S, Bessi VL, Mofarrahi M, Hussain SN, et al (2013). Protective role of PARK2/Parkin in sepsis-induced cardiac contractile and mitochondrial dysfunction. Autophagy, 9:1837-1851. 24121678 10.4161/auto.26502

[83] Li J, Shi W, Zhang J, Ren L (2019). To Explore the Protective Mechanism of PTEN-Induced Kinase 1 (PINK1)/Parkin Mitophagy-Mediated Extract of Periplaneta Americana on Lipopolysaccharide-Induced Cardiomyocyte Injury. Med Sci Monit, 25:1383-1391. 30789157 10.12659/MSM.912980 PMC6394139

[84] Eisenberg T, Abdellatif M, Schroeder S, Primessnig U, Stekovic S, Pendl T, et al (2016). Cardioprotection and lifespan extension by the natural polyamine spermidine. Nat Med, 22:1428-1438. 27841876 10.1038/nm.4222 PMC5806691

[85] Chen H, Ren S, Clish C, Jain M, Mootha V, McCaffery JM, et al (2015). Titration of mitochondrial fusion rescues Mff-deficient cardiomyopathy. J Cell Biol, 211:795-805. 26598616 10.1083/jcb.201507035 PMC4657172

[86] Hoshino A, Mita Y, Okawa Y, Ariyoshi M, Iwai-Kanai E, Ueyama T, et al (2013). Cytosolic p53 inhibits Parkin-mediated mitophagy and promotes mitochondrial dysfunction in the mouse heart. Nat Commun, 4:2308. 23917356 10.1038/ncomms3308

[87] Bell RM, Yellon DM (2011). There is more to life than revascularization: therapeutic targeting of myocardial ischemia/reperfusion injury. Cardiovasc Ther, 29:e67-79. 20645988 10.1111/j.1755-5922.2010.00190.x

[88] Moran AE, Forouzanfar MH, Roth GA, Mensah GA, Ezzati M, Murray CJ, et al (2014). Temporal trends in ischemic heart disease mortality in 21 world regions, 1980 to 2010: the Global Burden of Disease 2010 study. Circulation, 129:1483-1492. 24573352 10.1161/CIRCULATIONAHA.113.004042 PMC4181359

[89] Chouchani ET, Pell VR, Gaude E, Aksentijevic D, Sundier SY, Robb EL, et al (2014). Ischaemic accumulation of succinate controls reperfusion injury through mitochondrial ROS. Nature, 515:431-435. 25383517 10.1038/nature13909 PMC4255242

[90] Lesnefsky EJ, Chen Q, Tandler B, Hoppel CL (2017). Mitochondrial Dysfunction and Myocardial Ischemia-Reperfusion: Implications for Novel Therapies. Annu Rev Pharmacol Toxicol, 57:535-565. 27860548 10.1146/annurev-pharmtox-010715-103335 PMC11060135

[91] Li Y, Liu X (2018). Novel insights into the role of mitochondrial fusion and fission in cardiomyocyte apoptosis induced by ischemia/reperfusion. J Cell Physiol, 233:5589-5597. 29528108 10.1002/jcp.26522

[92] Kubli DA, Gustafsson AB (2012). Mitochondria and mitophagy: the yin and yang of cell death control. Circ Res, 111:1208-1221. 23065344 10.1161/CIRCRESAHA.112.265819 PMC3538875

[93] Feng Y, Madungwe NB, da Cruz Junho CV, Bopassa JC (2017). Activation of G protein-coupled oestrogen receptor 1 at the onset of reperfusion protects the myocardium against ischemia/reperfusion injury by reducing mitochondrial dysfunction and mitophagy. Br J Pharmacol, 174:4329-4344. 28906548 10.1111/bph.14033 PMC5715577

[94] Li YZ, Wu XD, Liu XH, Li PF (2018). Mitophagy imbalance in cardiomyocyte ischaemia/reperfusion injury. Acta Physiol (Oxf), 225:e13228. 30507035 10.1111/apha.13228

[95] Liu XW, Lu MK, Zhong HT, Wang LH, Fu YP (2019). Panax Notoginseng Saponins Attenuate Myocardial Ischemia-Reperfusion Injury Through the HIF-1alpha/BNIP3 Pathway of Autophagy. J Cardiovasc Pharmacol, 73:92-99. 30531436 10.1097/FJC.0000000000000640

[96] Zhang W, Siraj S, Zhang R, Chen Q (2017). Mitophagy receptor FUNDC1 regulates mitochondrial homeostasis and protects the heart from I/R injury. Autophagy, 13:1080-1081. 28323531 10.1080/15548627.2017.1300224 PMC5486361

[97] Hamacher-Brady A, Brady NR, Logue SE, Sayen MR, Jinno M, Kirshenbaum LA, et al (2007). Response to myocardial ischemia/reperfusion injury involves Bnip3 and autophagy. Cell Death Differ, 14:146-157. 16645637 10.1038/sj.cdd.4401936

[98] Jin Q, Li R, Hu N, Xin T, Zhu P, Hu S, et al (2018). DUSP1 alleviates cardiac ischemia/reperfusion injury by suppressing the Mff-required mitochondrial fission and Bnip3-related mitophagy via the JNK pathways. Redox Biol, 14:576-587. 29149759 10.1016/j.redox.2017.11.004 PMC5691221

[99] Yu W, Xu M, Zhang T, Zhang Q, Zou C (2019). Mst1 promotes cardiac ischemia-reperfusion injury by inhibiting the ERK-CREB pathway and repressing FUNDC1-mediated mitophagy. J Physiol Sci, 69:113-127. 29961191 10.1007/s12576-018-0627-3 PMC10717665

[100] Galloway CA, Yoon Y (2015). Mitochondrial dynamics in diabetic cardiomyopathy. Antioxid Redox Signal, 22:1545-1562. 25738230 10.1089/ars.2015.6293 PMC4449634

[101] Nishikawa T, Edelstein D, Du XL, Yamagishi S, Matsumura T, Kaneda Y, et al (2000). Normalizing mitochondrial superoxide production blocks three pathways of hyperglycaemic damage. Nature, 404:787-790. 10783895 10.1038/35008121

[102] Frustaci A, Kajstura J, Chimenti C, Jakoniuk I, Leri A, Maseri A, et al (2000). Myocardial cell death in human diabetes. Circ Res, 87:1123-1132. 11110769 10.1161/01.res.87.12.1123

[103] Shen X, Zheng S, Metreveli NS, Epstein PN (2006). Protection of cardiac mitochondria by overexpression of MnSOD reduces diabetic cardiomyopathy. Diabetes, 55:798-805. 16505246 10.2337/diabetes.55.03.06.db05-1039

[104] Zorov DB, Juhaszova M, Sollott SJ (2014). Mitochondrial reactive oxygen species (ROS) and ROS-induced ROS release. Physiol Rev, 94:909-950. 24987008 10.1152/physrev.00026.2013 PMC4101632

[105] Huynh K, Bernardo BC, McMullen JR, Ritchie RH (2014). Diabetic cardiomyopathy: mechanisms and new treatment strategies targeting antioxidant signaling pathways. Pharmacol Ther, 142:375-415. 24462787 10.1016/j.pharmthera.2014.01.003

[106] Johansen JS, Harris AK, Rychly DJ, Ergul A (2005). Oxidative stress and the use of antioxidants in diabetes: linking basic science to clinical practice. Cardiovasc Diabetol, 4:5. 15862133 10.1186/1475-2840-4-5 PMC1131912

[107] Shen X, Zheng S, Thongboonkerd V, Xu M, Pierce WM Jr, Klein JB, et al (2004). Cardiac mitochondrial damage and biogenesis in a chronic model of type 1 diabetes. Am J Physiol Endocrinol Metab, 287:E896-905. 15280150 10.1152/ajpendo.00047.2004

[108] Tomita M, Mukae S, Geshi E, Umetsu K, Nakatani M, Katagiri T (1996). Mitochondrial respiratory impairment in streptozotocin-induced diabetic rat heart. Jpn Circ J, 60:673-682. 8902585 10.1253/jcj.60.673

[109] Xu X, Kobayashi S, Chen K, Timm D, Volden P, Huang Y, et al (2013). Diminished autophagy limits cardiac injury in mouse models of type 1 diabetes. J Biol Chem, 288:18077-18092. 23658055 10.1074/jbc.M113.474650 PMC3689952

[110] Mellor KM, Reichelt ME, Delbridge LM (2011). Autophagy anomalies in the diabetic myocardium. Autophagy, 7:1263-1267. 21814044 10.4161/auto.7.10.17148

[111] Mellor KM, Bell JR, Young MJ, Ritchie RH, Delbridge LM (2011). Myocardial autophagy activation and suppressed survival signaling is associated with insulin resistance in fructose-fed mice. J Mol Cell Cardiol, 50:1035-1043. 21385586 10.1016/j.yjmcc.2011.03.002

[112] Xie Z, Lau K, Eby B, Lozano P, He C, Pennington B, et al (2011). Improvement of cardiac functions by chronic metformin treatment is associated with enhanced cardiac autophagy in diabetic OVE26 mice. Diabetes, 60:1770-1778. 21562078 10.2337/db10-0351 PMC3114402

[113] Durga Devi T, Babu M, Makinen P, Kaikkonen MU, Heinaniemi M, Laakso H, et al (2017). Aggravated Postinfarct Heart Failure in Type 2 Diabetes Is Associated with Impaired Mitophagy and Exaggerated Inflammasome Activation. Am J Pathol, 187:2659-2673. 28935571 10.1016/j.ajpath.2017.08.023

[114] Koncsos G, Varga ZV, Baranyai T, Boengler K, Rohrbach S, Li L, et al (2016). Diastolic dysfunction in prediabetic male rats: Role of mitochondrial oxidative stress. Am J Physiol Heart Circ Physiol, 311:H927-h943. 27521417 10.1152/ajpheart.00049.2016 PMC5114470

[115] Scheele C, Nielsen AR, Walden TB, Sewell DA, Fischer CP, Brogan RJ, et al (2007). Altered regulation of the PINK1 locus: a link between type 2 diabetes and neurodegeneration? Faseb j, 21:3653-3665. 17567565 10.1096/fj.07-8520com

[116] Tang Y, Liu J, Long J (2015). Phosphatase and tensin homolog-induced putative kinase 1 and Parkin in diabetic heart: Role of mitophagy. J Diabetes Investig, 6:250-255. 10.1111/jdi.12302 PMC4420554 25969707

[117] Wu W, Xu H, Wang Z, Mao Y, Yuan L, Luo W, et al (2015). PINK1-Parkin-Mediated Mitophagy Protects Mitochondrial Integrity and Prevents Metabolic Stress-Induced Endothelial Injury. PLoS One, 10:e0132499. 26161534 10.1371/journal.pone.0132499 PMC4498619

[118] Mozaffarian D, Benjamin EJ, Go AS, Arnett DK, Blaha MJ, Cushman M, et al (2016). Heart Disease and Stroke Statistics-2016 Update: A Report From the American Heart Association. Circulation, 133:e38-360. 26673558 10.1161/CIR.0000000000000350

[119] Grieve DJ, Shah AM (2003). Oxidative stress in heart failure. More than just damage. Eur Heart J, 24:2161-2163. 14659766 10.1016/j.ehj.2003.10.015

[120] von Hardenberg A, Maack C (2017). Mitochondrial Therapies in Heart Failure. Handb Exp Pharmacol, 243:491-514. 28181010 10.1007/164_2016_123

[121] Rosca MG, Hoppel CL (2013). Mitochondrial dysfunction in heart failure. Heart Fail Rev, 18:607-622. 22948484 10.1007/s10741-012-9340-0 PMC3855291

[122] Shires SE, Gustafsson AB (2015). Mitophagy and heart failure. J Mol Med (Berl), 93:253-262. 25609139 10.1007/s00109-015-1254-6 PMC4334711

[123] Qiu Z, Hu Y, Geng Y, Wu H, Bo R, Shi J, et al (2018). Xin Fu Kang oral liquid inhibits excessive myocardial mitophagy in a rat model of advanced heart failure. Am J Transl Res, 10:3198-3210. 30416661 PMC6220223

[124] Kassiotis C, Ballal K, Wellnitz K, Vela D, Gong M, Salazar R, et al (2009). Markers of autophagy are downregulated in failing human heart after mechanical unloading. Circulation, 120:S191-197. 19752367 10.1161/CIRCULATIONAHA.108.842252 PMC2778323

[125] Mori J, Zhang L, Oudit GY, Lopaschuk GD (2013). Impact of the renin-angiotensin system on cardiac energy metabolism in heart failure. J Mol Cell Cardiol, 63:98-106. 23886814 10.1016/j.yjmcc.2013.07.010

[126] Wang B, Nie J, Wu L, Hu Y, Wen Z, Dong L, et al (2018). AMPKalpha2 Protects Against the Development of Heart Failure by Enhancing Mitophagy via PINK1 Phosphorylation. Circ Res, 122:712-729. 29284690 10.1161/CIRCRESAHA.117.312317 PMC5834386

[127] Shires SE, Gustafsson AB (2018). Regulating Renewable Energy: Connecting AMPKalpha2 to PINK1/Parkin-Mediated Mitophagy in the Heart. Circ Res, 122:649-651. 29496793 10.1161/CIRCRESAHA.118.312655 PMC5836749

[128] Billia F, Hauck L, Konecny F, Rao V, Shen J, Mak TW (2011). PTEN-inducible kinase 1 (PINK1)/Park6 is indispensable for normal heart function. Proc Natl Acad Sci U S A, 108:9572-9577. 21606348 10.1073/pnas.1106291108 PMC3111326

[129] Diwan A, Wansapura J, Syed FM, Matkovich SJ, Lorenz JN, Dorn GW 2nd, (2008). Nix-mediated apoptosis links myocardial fibrosis, cardiac remodeling, and hypertrophy decompensation. Circulation, 117:396-404. 18178777 10.1161/CIRCULATIONAHA.107.727073 PMC2538800

[130] Gakidou E, Afshin A, Abajobir A, Abate K, Abbafati C, Abbas K, et al (2018). Global, regional, and national comparative risk assessment of 84 behavioural, environmental and occupational, and metabolic risks or clusters of risks for 195 countries and territories, 1990-2017: a systematic analysis for the Global Burden of Disease Study 2017. Lancet, 392:1923-1994. 30496105 10.1016/S0140-6736(18)32225-6 PMC6227755

[131] Lahera V, de Las Heras N, Lopez-Farre A, Manucha W, Ferder L (2017). Role of Mitochondrial Dysfunction in Hypertension and Obesity. Curr Hypertens Rep, 19:11. 28233236 10.1007/s11906-017-0710-9

[132] Dikalov SI, Dikalova A (2019). Crosstalk between mitochondrial hyperacetylation and oxidative stress in vascular dysfunction and hypertension. Antioxid Redox Signal. 10.1089/ars.2018.7632 PMC6708267 30618267

[133] Dikalov S, Itani HA, Richmond B, Vergeade A, Rahman SMJ, Boutaud O, et al (2019). Tobacco Smoking Induces Cardiovascular Mitochondrial Oxidative Stress, Promotes Endothelial Dysfunction and Enhances Hypertension. Am J Physiol Heart Circ Physiol, 316:H639-h646. 30608177 10.1152/ajpheart.00595.2018 PMC6459311

[134] Smolders VF, Zodda E, Quax PHA, Carini M, Barbera JA, Thomson TM, et al (2018). Metabolic Alterations in Cardiopulmonary Vascular Dysfunction. Front Mol Biosci, 5:120. 30723719 10.3389/fmolb.2018.00120 PMC6349769

[135] Neubauer S (2007). The failing heart--an engine out of fuel. N Engl J Med, 356:1140-1151. 17360992 10.1056/NEJMra063052

[136] Togliatto G, Lombardo G, Brizzi MF (2017). The Future Challenge of Reactive Oxygen Species (ROS) in Hypertension: From Bench to Bed Side. Int J Mol Sci, 18:E1988. 28914782 10.3390/ijms18091988 PMC5618637

[137] Fang L, Moore XL, Gao XM, Dart AM, Lim YL, Du XJ (2007). Down-regulation of mitofusin-2 expression in cardiac hypertrophy in vitro and in vivo. Life Sci, 80:2154-2160. 17499311 10.1016/j.lfs.2007.04.003

[138] Ashrafian H, Docherty L, Leo V, Towlson C, Neilan M, Steeples V, et al (2010). A mutation in the mitochondrial fission gene Dnm1l leads to cardiomyopathy. PLoS Genet, 6:e1001000. 20585624 10.1371/journal.pgen.1001000 PMC2891719

[139] Wu MY, Li CJ, Hou MF, Chu PY (2017). New Insights into the Role of Inflammation in the Pathogenesis of Atherosclerosis. Int J Mol Sci, 18:E2034. 28937652 10.3390/ijms18102034 PMC5666716

[140] Clarke MC, Littlewood TD, Figg N, Maguire JJ, Davenport AP, Goddard M, et al (2008). Chronic apoptosis of vascular smooth muscle cells accelerates atherosclerosis and promotes calcification and medial degeneration. Circ Res, 102:1529-1538. 18497329 10.1161/CIRCRESAHA.108.175976

[141] Hansson GK, Hermansson A (2011). The immune system in atherosclerosis. Nat Immunol, 12:204-212. 21321594 10.1038/ni.2001

[142] Shioi A, Ikari Y (2018). Plaque Calcification During Atherosclerosis Progression and Regression. J Atheroscler Thromb, 25:294-303. 29238011 10.5551/jat.RV17020 PMC5906181

[143] Grootaert MOJ, Roth L, Schrijvers DM, De Meyer GRY, Martinet W (2018). Defective Autophagy in Atherosclerosis: To Die or to Senesce? Oxid Med Cell Longev, 2018:7687083. 29682164 10.1155/2018/7687083 PMC5846382

[144] Kattoor AJ, Pothineni NVK, Palagiri D, Mehta JL (2017). Oxidative Stress in Atherosclerosis. Curr Atheroscler Rep, 19:42. 28921056 10.1007/s11883-017-0678-6

[145] Li P, Bai Y, Zhao X, Tian T, Tang L, Ru J, et al (2018). NR4A1 contributes to high-fat associated endothelial dysfunction by promoting CaMKII-Parkin-mitophagy pathways. Cell Stress Chaperones, 23:749-761. 29470798 10.1007/s12192-018-0886-1 PMC6045535

[146] Swiader A, Nahapetyan H, Faccini J, D'Angelo R, Mucher E, Elbaz M, et al (2016). Mitophagy acts as a safeguard mechanism against human vascular smooth muscle cell apoptosis induced by atherogenic lipids. Oncotarget, 7:28821-28835. 27119505 10.18632/oncotarget.8936 PMC5045359

[147] Docherty CK, Carswell A, Friel E, Mercer JR (2018). Impaired mitochondrial respiration in human carotid plaque atherosclerosis: A potential role for Pink1 in vascular smooth muscle cell energetics. Atherosclerosis, 268:1-11. 29156421 10.1016/j.atherosclerosis.2017.11.009 PMC6565844

[148] Bravo-San Pedro JM, Kroemer G, Galluzzi L (2017). Autophagy and Mitophagy in Cardiovascular Disease. Circ Res, 120:1812-1824. 28546358 10.1161/CIRCRESAHA.117.311082

[149] Santulli G, Iaccarino G, De Luca N, Trimarco B, Condorelli G (2014). Atrial fibrillation and microRNAs. Front Physiol, 5:15. 24478726 10.3389/fphys.2014.00015 PMC3900852

[150] Yang KC, Bonini MG, Dudley SC Jr, (2014). Mitochondria and arrhythmias. Free Radic Biol Med, 71:351-361. 24713422 10.1016/j.freeradbiomed.2014.03.033 PMC4096785

[151] Wirth KJ, Rosenstein B, Uhde J, Englert HC, Busch AE, Scholkens BA (1999). ATP-sensitive potassium channel blocker HMR 1883 reduces mortality and ischemia-associated electrocardiographic changes in pigs with coronary occlusion. J Pharmacol Exp Ther, 291:474-481. 10525061

[152] Brennan JP, Southworth R, Medina RA, Davidson SM, Duchen MR, Shattock MJ (2006). Mitochondrial uncoupling, with low concentration FCCP, induces ROS-dependent cardioprotection independent of KATP channel activation. Cardiovasc Res, 72:313-321. 16950237 10.1016/j.cardiores.2006.07.019

[153] Gambardella J, Sorriento D, Ciccarelli M, Del Giudice C, Fiordelisi A, Napolitano L, et al (2017). Functional Role of Mitochondria in Arrhythmogenesis. Adv Exp Med Biol, 982:191-202. 28551788 10.1007/978-3-319-55330-6_10 PMC6709870

[154] Xi G, Keep RF, Hoff JT (2006). Mechanisms of brain injury after intracerebral haemorrhage. Lancet Neurol, 5:53-63. 16361023 10.1016/S1474-4422(05)70283-0

[155] Kuramatsu JB, Huttner HB, Schwab S (2013). Advances in the management of intracerebral hemorrhage. J Neural Transm (Vienna), 120 Suppl 1:S35-41. 23720189 10.1007/s00702-013-1040-y

[156] Manno EM, Atkinson JL, Fulgham JR, Wijdicks EF (2005). Emerging medical and surgical management strategies in the evaluation and treatment of intracerebral hemorrhage. Mayo Clin Proc, 80:420-433. 15757025 10.4065/80.3.420

[157] Jin Z, Wu J, Yan LJ (2016). Chemical Conditioning as an Approach to Ischemic Stroke Tolerance: Mitochondria as the Target. Int J Mol Sci, 17:351. 27005615 10.3390/ijms17030351 PMC4813212

[158] Watts LT, Lloyd R, Garling RJ, Duong T (2013). Stroke neuroprotection: targeting mitochondria. Brain Sci, 3:540-560. 24961414 10.3390/brainsci3020540 PMC4061853

[159] Kim-Han JS, Kopp SJ, Dugan LL, Diringer MN (2006). Perihematomal mitochondrial dysfunction after intracerebral hemorrhage. Stroke, 37:2457-2462. 16960094 10.1161/01.STR.0000240674.99945.4e

[160] Shi RY, Zhu SH, Li V, Gibson SB, Xu XS, Kong JM (2014). BNIP3 interacting with LC3 triggers excessive mitophagy in delayed neuronal death in stroke. CNS Neurosci Ther, 20:1045-1055. 25230377 10.1111/cns.12325 PMC6492992

[161] Yuan Y, Zheng Y, Zhang X, Chen Y, Wu X, Wu J, et al (2017). BNIP3L/NIX-mediated mitophagy protects against ischemic brain injury independent of PARK2. Autophagy, 13:1754-1766. 28820284 10.1080/15548627.2017.1357792 PMC5640199

[162] Lan R, Wu JT, Wu T, Ma YZ, Wang BQ, Zheng HZ, et al (2018). Mitophagy is activated in brain damage induced by cerebral ischemia and reperfusion via the PINK1/Parkin/p62 signalling pathway. Brain Res Bull, 142:63-77. 29964088 10.1016/j.brainresbull.2018.06.018

[163] Feng J, Chen X, Shen J (2017). Reactive nitrogen species as therapeutic targets for autophagy: implication for ischemic stroke. Expert Opin Ther Targets, 21:305-317. 28081644 10.1080/14728222.2017.1281250

[164] Mohr AM, Mott JL (2015). Overview of microRNA biology. Semin Liver Dis, 35:3-11. 25632930 10.1055/s-0034-1397344 PMC4797991

[165] Yang F, Li T, Dong Z, Mi R (2018). MicroRNA-410 is involved in mitophagy after cardiac ischemia/reperfusion injury by targeting high-mobility group box 1 protein. J Cell Biochem, 119:2427-2439. 28914970 10.1002/jcb.26405

[166] Li W, Zhang X, Zhuang H, Chen HG, Chen Y, Tian W, et al (2014). MicroRNA-137 is a novel hypoxia-responsive microRNA that inhibits mitophagy via regulation of two mitophagy receptors FUNDC1 and NIX. J Biol Chem, 289:10691-10701. 24573672 10.1074/jbc.M113.537050 PMC4036186

[167] Mughal W, Nguyen L, Pustylnik S, da Silva Rosa SC, Piotrowski S, Chapman D, et al (2015). A conserved MADS-box phosphorylation motif regulates differentiation and mitochondrial function in skeletal, cardiac, and smooth muscle cells. Cell Death Dis, 6:e1944. 26512955 10.1038/cddis.2015.306 PMC5399178

[168] Zhou H, Zhang Y, Hu S, Shi C, Zhu P, Ma Q, et al (2017). Melatonin protects cardiac microvasculature against ischemia/reperfusion injury via suppression of mitochondrial fission-VDAC1-HK2-mPTP-mitophagy axis. J Pineal Res, 63:e12413. 10.1111/jpi.12413 PMC5518188 28398674

[169] Wang S, Zhao Z, Feng X, Cheng Z, Xiong Z, Wang T, et al (2018). Melatonin activates Parkin translocation and rescues the impaired mitophagy activity of diabetic cardiomyopathy through Mst1 inhibition. J Cell Mol Med, 22:5132-5144. 30063115 10.1111/jcmm.13802 PMC6156356

[170] Ma S, Chen J, Feng J, Zhang R, Fan M, Han D, et al (2018). Melatonin Ameliorates the Progression of Atherosclerosis via Mitophagy Activation and NLRP3 Inflammasome Inhibition. Oxid Med Cell Longev, 2018:9286458. 10.1155/2018/9286458 PMC6142770 30254716

[171] Andres AM, Hernandez G, Lee P, Huang C, Ratliff EP, Sin J, et al (2014). Mitophagy is required for acute cardioprotection by simvastatin. Antioxid Redox Signal, 21:1960-1973. 23901824 10.1089/ars.2013.5416 PMC4208607

[172] Qiao H, Ren H, Du H, Zhang M, Xiong X, Lv R (2018). Liraglutide repairs the infarcted heart: The role of the SIRT1/Parkin/mitophagy pathway. Mol Med Rep, 17:3722-3734. 29328405 10.3892/mmr.2018.8371 PMC5802177

[173] Bian X, Teng T, Zhao H, Qin J, Qiao Z, Sun Y, et al (2018). Zinc prevents mitochondrial superoxide generation by inducing mitophagy in the setting of hypoxia/reoxygenation in cardiac cells. Free Radic Res, 52:80-91. 29216769 10.1080/10715762.2017.1414949

[174] Yu P, Zhang J, Yu S, Luo Z, Hua F, Yuan L, et al (2015). Protective Effect of Sevoflurane Postconditioning against Cardiac Ischemia/Reperfusion Injury via Ameliorating Mitochondrial Impairment, Oxidative Stress and Rescuing Autophagic Clearance. PLoS One, 10:e0134666. 26263161 10.1371/journal.pone.0134666 PMC4532466

[175] Ma L, Zhu J, Gao Q, Rebecchi MJ, Wang Q, Liu L (2017). Restoring Pharmacologic Preconditioning in the Aging Heart: Role of Mitophagy/Autophagy. J Gerontol A Biol Sci Med Sci, 72:489-498. 27565512 10.1093/gerona/glw168

[176] Thompson JW, Wei J, Appau K, Wang H, Yu H, Spiga MG, et al (2015). Bnip3 Binds and Activates p300: Possible Role in Cardiac Transcription and Myocyte Morphology. PLoS One, 10:e0136847. 26317696 10.1371/journal.pone.0136847 PMC4552727

[177] Dhingra A, Jayas R, Afshar P, Guberman M, Maddaford G, Gerstein J, et al (2017). Ellagic acid antagonizes Bnip3-mediated mitochondrial injury and necrotic cell death of cardiac myocytes. Free Radic Biol Med, 112:411-422. 28838842 10.1016/j.freeradbiomed.2017.08.010

[178] Zhang X, Yuan Y, Jiang L, Zhang J, Gao J, Shen Z, et al (2014). Endoplasmic reticulum stress induced by tunicamycin and thapsigargin protects against transient ischemic brain injury: Involvement of PARK2-dependent mitophagy. Autophagy, 10:1801-1813. 25126734 10.4161/auto.32136 PMC4198364

[179] Feng J, Chen X, Guan B, Li C, Qiu J, Shen J (2018). Inhibition of Peroxynitrite-Induced Mitophagy Activation Attenuates Cerebral Ischemia-Reperfusion Injury. Mol Neurobiol, 55:6369-6386. 29307080 10.1007/s12035-017-0859-x

[180] Feng J, Chen X, Lu S, Li W, Yang D, Su W, et al (2018). Naringin Attenuates Cerebral Ischemia-Reperfusion Injury Through Inhibiting Peroxynitrite-Mediated Mitophagy Activation. Mol Neurobiol, 55:9029-9042. 29627876 10.1007/s12035-018-1027-7

[181] Zhang Z, Yu J (2018). NR4A1 Promotes Cerebral Ischemia Reperfusion Injury by Repressing Mfn2-Mediated Mitophagy and Inactivating the MAPK-ERK-CREB Signaling Pathway. Neurochem Res, 43:1963-1977. 30136162 10.1007/s11064-018-2618-4

[182] Hammerling BC, Najor RH, Cortez MQ, Shires SE, Leon LJ, Gonzalez ER, et al (2017). A Rab5 endosomal pathway mediates Parkin-dependent mitochondrial clearance. Nat Commun, 8:14050. 28134239 10.1038/ncomms14050 PMC5290275

[183] Hoshino A, Matoba S, Iwai-Kanai E, Nakamura H, Kimata M, Nakaoka M, et al (2012). p53-TIGAR axis attenuates mitophagy to exacerbate cardiac damage after ischemia. J Mol Cell Cardiol, 52:175-184. 22044588 10.1016/j.yjmcc.2011.10.008

[184] Chaanine AH, Jeong D, Liang L, Chemaly ER, Fish K, Gordon RE, et al (2012). JNK modulates FOXO3a for the expression of the mitochondrial death and mitophagy marker BNIP3 in pathological hypertrophy and in heart failure. Cell Death Dis, 3:265. 22297293 10.1038/cddis.2012.5 PMC3288347

[185] Bourke LT, Knight RA, Latchman DS, Stephanou A, McCormick J (2013). Signal transducer and activator of transcription-1 localizes to the mitochondria and modulates mitophagy. Jakstat, 2:e25666. 24470977 10.4161/jkst.25666 PMC3902047

[186] Ji W, Wei S, Hao P, Xing J, Yuan Q, Wang J, et al (2016). Aldehyde Dehydrogenase 2 Has Cardioprotective Effects on Myocardial Ischaemia/Reperfusion Injury via Suppressing Mitophagy. Front Pharmacol, 7:101. 27148058 10.3389/fphar.2016.00101 PMC4838626

[187] Yu W, Gao B, Li N, Wang J, Qiu C, Zhang G, et al (2017). Sirt3 deficiency exacerbates diabetic cardiac dysfunction: Role of Foxo3A-Parkin-mediated mitophagy. Biochim Biophys Acta Mol Basis Dis, 1863:1973-1983. 27794418 10.1016/j.bbadis.2016.10.021

[188] Wang S, Zhao Z, Fan Y, Zhang M, Feng X, Lin J, et al (2018). Mst1 inhibits Sirt3 expression and contributes to diabetic cardiomyopathy through inhibiting Parkin-dependent mitophagy. Biochim Biophys Acta Mol Basis Dis, 18:S0925-4439. 10.1016/j.bbadis.2018.04.009 29674007

[189] Tahrir FG, Knezevic T, Gupta MK, Gordon J, Cheung JY, Feldman AM, et al (2017). Evidence for the Role of BAG3 in Mitochondrial Quality Control in Cardiomyocytes. J Cell Physiol, 232:797-805. 27381181 10.1002/jcp.25476 PMC5663276

[190] He L, Zhou Q, Huang Z, Xu J, Zhou H, Lv D, et al (2018). PINK1/Parkin-mediated mitophagy promotes apelin-13-induced vascular smooth muscle cell proliferation by AMPKalpha and exacerbates atherosclerotic lesions. J Cell Physiol, 234:8668-8682. 30456860 10.1002/jcp.27527

[191] Zha Z, Wang J, Wang X, Lu M, Guo Y (2017). Involvement of PINK1/Parkin-mediated mitophagy in AGE-induced cardiomyocyte aging. Int J Cardiol, 227:201-208. 27839819 10.1016/j.ijcard.2016.11.161

[192] Ren J, Yang L, Zhu L, Xu X, Ceylan AF, Guo W, et al (2017). Akt2 ablation prolongs life span and improves myocardial contractile function with adaptive cardiac remodeling: role of Sirt1-mediated autophagy regulation. Aging Cell, 16:976-987. 28681509 10.1111/acel.12616 PMC5595687

[193] Bultman SJ, Holley DW, G GdR, Pizzo SV, Sidorova TN, Murray KT, et al (2016). BRG1 and BRM SWI/SNF ATPases redundantly maintain cardiomyocyte homeostasis by regulating cardiomyocyte mitophagy and mitochondrial dynamics in vivo. Cardiovasc Pathol, 25:258-269. 27039070 10.1016/j.carpath.2016.02.004 PMC4860071

[194] Lu J, Qian HY, Liu LJ, Zhou BC, Xiao Y, Mao JN, et al (2014). Mild hypothermia alleviates excessive autophagy and mitophagy in a rat model of asphyxial cardiac arrest. Neurol Sci, 35:1691-1699. 24816750 10.1007/s10072-014-1813-6

[195] Yuan Y, Pan SS (2018). Parkin Mediates Mitophagy to Participate in Cardioprotection Induced by Late Exercise Preconditioning but Bnip3 Does Not. J. Cardiovasc. Pharmacol., 71:303-316. 10.1097/FJC.0000000000000572 29538088

[196] Shen Z, Zheng Y, Wu J, Chen Y, Wu X, Zhou Y, et al (2017). PARK2-dependent mitophagy induced by acidic postconditioning protects against focal cerebral ischemia and extends the reperfusion window. Autophagy, 13:473-485. 28103118 10.1080/15548627.2016.1274596 PMC5361599

[197] Zhou M, Xia ZY, Lei SQ, Leng Y, Xue R (2015). Role of mitophagy regulated by Parkin/DJ-1 in remote ischemic postconditioning-induced mitigation of focal cerebral ischemia-reperfusion. Eur Rev Med Pharmacol Sci, 19:4866-4871. 26744879

